# Clinical response to nivolumab in an INI1-deficient pediatric chordoma correlates with immunogenic recognition of brachyury

**DOI:** 10.1038/s41698-021-00238-4

**Published:** 2021-12-20

**Authors:** Laura M. Williamson, Craig M. Rive, Daniela Di Francesco, Emma Titmuss, Hye-Jung E. Chun, Scott D. Brown, Katy Milne, Erin Pleasance, Anna F. Lee, Stephen Yip, Daniel G. Rosenbaum, Martin Hasselblatt, Pascal D. Johann, Marcel Kool, Melissa Harvey, David Dix, Daniel J. Renouf, Robert A. Holt, Brad H. Nelson, Martin Hirst, Steven J. M. Jones, Janessa Laskin, Shahrad R. Rassekh, Rebecca J. Deyell, Marco A. Marra

**Affiliations:** 1 Canada’s Michael Smith Genome Sciences Centre at BC Cancer, Vancouver, BC Canada; 2 Deeley Research Centre, BC Cancer, Victoria, BC Canada; 3 Department of Biochemistry and Microbiology, University of Victoria, Victoria, BC Canada; 4 Department of Pathology and Laboratory Medicine, British Columbia Children’s Hospital, Vancouver, BC Canada; 5 Department of Pathology and Laboratory Medicine, Vancouver General Hospital, Vancouver, BC Canada; 6 Department of Radiology, British Columbia Children’s Hospital, Vancouver, BC Canada; 7 Institute of Neuropathology, University Hospital Münster, Münster, Germany; 8 Hopp Children’s Cancer Center (KITZ), Heidelberg, Germany; 9 Division of Pediatric Neurooncology, German Cancer Research Center (DKFZ) and German Cancer Consortium (DKTK) Core Center, Heidelberg, Germany; 10 Department of Pediatric Hematology and Oncology, University Hospital Heidelberg, Heidelberg, Germany; 11 Princess Máxima Center for Pediatric Oncology, Utrecht, the Netherlands; 12 Division of Pediatric Hematology Oncology BMT, University of British Columbia, Vancouver, BC Canada; 13 Pancreas Centre BC, Vancouver, BC Canada; 14 Department of Medical Oncology, BC Cancer, Vancouver, BC Canada; 15 Department of Medical Genetics, University of British Columbia, Vancouver, BC Canada; 16 Department of Molecular Biology and Biochemistry, Simon Fraser University, Burnaby, BC Canada; 17 Department of Microbiology & Immunology, Michael Smith Laboratories, University of British Columbia, Vancouver, BC Canada

**Keywords:** Predictive markers, Paediatric cancer, Sarcoma, Molecular medicine

## Abstract

Poorly differentiated chordoma (PDC) is a recently recognized subtype of chordoma characterized by expression of the embryonic transcription factor, brachyury, and loss of INI1. PDC primarily affects children and is associated with a poor prognosis and limited treatment options. Here we describe the molecular and immune tumour microenvironment profiles of two paediatric PDCs produced using whole-genome, transcriptome and whole-genome bisulfite sequencing (WGBS) and multiplex immunohistochemistry. Our analyses revealed the presence of tumour-associated immune cells, including CD8+ T cells, and expression of the immune checkpoint protein, PD-L1, in both patient samples. Molecular profiling provided the rationale for immune checkpoint inhibitor (ICI) therapy, which resulted in a clinical and radiographic response. A dominant T cell receptor (TCR) clone specific for a brachyury peptide–MHC complex was identified from bulk RNA sequencing, suggesting that targeting of the brachyury tumour antigen by tumour-associated T cells may underlie this clinical response to ICI. Correlative analysis with rhabdoid tumours, another INI1-deficient paediatric malignancy, suggests that a subset of tumours may share common immune phenotypes, indicating the potential for a therapeutically targetable subgroup of challenging paediatric cancers.

## Introduction

Chordomas are malignant neoplasms derived from notochord cells typically originating in the spine, sacrum or base of the skull, affecting both adults and children. Poorly differentiated chordomas (PDCs) are a recently recognized distinct subgroup of chordoma, which occurs predominantly in children, and is characterized by the expression of brachyury, an embryonic transcription factor encoded by the *TBXT* gene, and by loss of the SWI/SNF chromatin remodelling factor subunit, INI1, encoded by the *SMARCB1* gene^1–5^. These aggressive tumours are associated with a high risk of rapid local recurrence and distant metastasis and are generally poor candidates for primary resection, highlighting the need for effective systemic therapy.

Immune checkpoint inhibitors (ICIs) are now the standard of care for various adult malignancies associated with high tumour mutation burden (TMB) including lung cancer, melanoma and tumours deficient in mismatch repair^6^. Paediatric tumours are typically associated with low TMB compared to adult tumours, and emerging data from several prospective clinical trials have reported an overall low rate of response to ICIs in solid paediatric tumours^7–9^. Although broad response to ICIs among paediatric patients is lacking, growing evidence has shown responses to ICIs in tumours deficient in SWI/SNF chromatin remodelling genes, including but not limited to chordomas, rhabdoid tumours (RTs), and small cell carcinoma of the ovary hypercalcemic type tumours that affect paediatric and young adult populations^9–15^. This highlights the need to identify those patients most likely to benefit from ICIs and distinguish these from the broader paediatric population. The presence of infiltrating CD8+ T cells and expression of immune checkpoint genes in a subset of tumours is compatible with the notion that malignancies characterized by SWI/SNF deficiency may correlate with an immune hot phenotype and therapeutic response to ICIs^10,11,13,14,16,17^.

Here we describe the molecular and immune tumour microenvironment profiles of two paediatric PDCs using whole-genome, transcriptome and whole-genome bisulfite sequencing (WGBS) and multiplex immunohistochemistry (IHC) as part of the Personalized OncoGenomics (POG) programme (NCT02155621) at BC Cancer. Our analyses provided evidence for the presence of tumour-infiltrating CD8+ T cells, a brachyury tumour antigen-directed immune response and the rationale for ICI therapy that resulted in a clinical and radiographic response in a PDC patient treated with nivolumab. Analysis of methylation profiles from paediatric chordomas and SWI/SNF-deficient RTs revealed a correlation between paediatric chordoma and a subgroup of RTs characterized by CD8+ T cell infiltration, supporting the hypothesis that molecularly similar entities from different cancer types may also benefit from ICI therapy.

## Results

### Clinical presentation

**Fig. 1:**
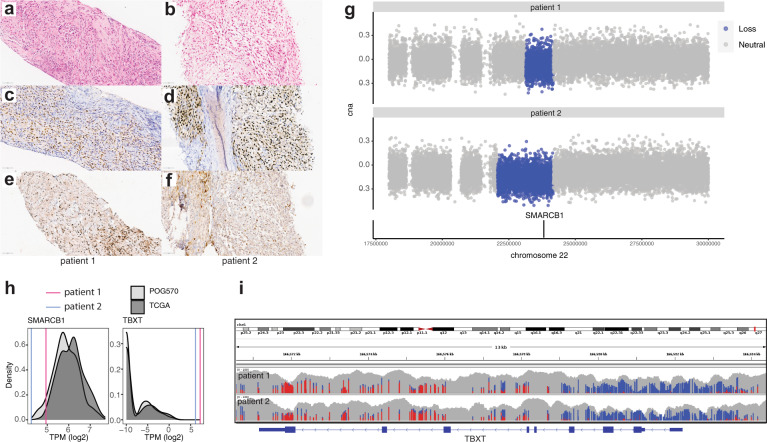
*SMARCB1*/INI1 loss and *TBXT*/brachyury expression in paediatric chordoma. Formalin-fixed paraffin-embedded (FFPE) sections of the biopsies from patient 1 (**a**, **c**, **e**) and patient 2 (**b**, **d**, **f**) display features consistent with a poorly differentiated chordoma (PDC) in these two patients. H&E photomicrographs (**a**, **b**) are composed of sheets of epithelioid to spindle cells associated with occasional eosinophilic cytoplasm and vesicular nuclei admixed with fibroblasts and inflammatory cells. Tumour cells strongly expressed nuclear brachyury (**c**, **d**) and are negative for nuclear expression of INI1 (**e**, **f**). Inflammatory cells retain nuclear expression of INI1 and are negative for brachyury expression. Original magnification: ×400, scale bars represent 60 μm. **g** Tumour/normal ratios of binned read depths and copy states for chromosome 22 (hg19 chr22:18000000–30000000) for patients 1 (top) and 2 (bottom). Regions defined as single copy loss are denoted in blue. The *SMARCB1* gene location is indicated by the black bar on the lower track. **h** Comparison of *SMARCB1* and *TBXT* gene expression in paediatric PDC to the TCGA sarcoma data set^18^ (dark grey, *n* = 261) and POG570 pan-cancer cohort (light grey, *n* = 570). Log2-transformed TPM is plotted on the *x*-axis and sample densities on the *y*-axis. The gene expression of *SMARCB1* and *TBXT* in paediatric chordomas is represented by vertical lines (patient 1; 4.94 and 7.2 log_2_(TPM) and patient 2; 4.26 and 6.1 log_2_(TPM), respectively). Median *SMARCB1* expression was 5.82 and 6.13 log_2_(TPM) for the POG570 and TCGA sarcoma cohorts, respectively. Median *TBXT* expression was −9.97 log_2_(TPM) for both POG570 and TCGA sarcoma cohorts. **i** DNA methylation profiles of the *TBXT* gene. Unmethylated (blue) and methylated (red) CpGs are shown for patients 1 and 2. The grey bars represent overall read depth (data range 0–100, log scale).

Patient 1 presented with worsening stiffness and pain of the neck. A magnetic resonance imaging (MRI) showed an infiltrating but non-enhancing soft tissue mass around the anterior atlantoaxial joint causing erosion of the bone and extending from the clivus into the prevertebral space to the right of C2. A computed tomographic (CT) scan showed bony erosion of both C1 and C2 and subsequent positron emission tomographic (PET) imaging revealed a fluorodeoxyglucose (FDG)-avid soft tissue mass of the neck (SUVmax 7.15) and left hilar adenopathy (SUVmax 2.34). Biopsy of the primary mass revealed an INI1-negative spindle cell neoplasm. A second biopsy was consistent with a diagnosis of an epithelioid sarcoma, with morphology more consistent with the conventional rather than the proximal type, with absence of overt rhabdoid morphology. Positive nuclear brachyury staining, strong keratin staining and negativity for S100 and INI1 supported a diagnosis of PDC (Fig. 1a, c, d and Supplementary Fig. 1). Patient 1 was treated with neoadjuvant vincristine, doxorubicin and cyclophosphamide with alternating ifosfamide and etoposide with good response, followed by high-dose proton beam radiation. The patient recurred with metastatic disease of the lung 3 months following radiation, and it was this metastatic site that was biopsied for whole-genome and transcriptome analysis (WGTA). Three additional cycles of chemotherapy were given with mixed response, and the patient died within a month of progressive disease.

Patient 2 presented with a 3-year history of recurrent headaches, followed by worsening neck discomfort and limited range of movement. An MRI of her head and neck revealed a large, multi-lobulated expansile soft tissue mass centred at the left anterior arch and lateral mass of the C1 vertebral body, measuring 4.3 × 2.1 × 2.8 cm and involving the clivus, occipital condyle and dens of C2. There was mass effect on the adjacent left vertebral artery and right anterior medulla, as well as on the tectorial membrane and para-pharyngeal space. Chest CT showed two irregular pleural-based densities that were resected and consistent with metastatic disease. A PET scan showed an FDG-avid mass at C1 involving the lateral mass, the dens, the clivus and the left occipital condyle. Two faintly avid densities were noted in the right lung, with lower PET avidity than would be expected of metastases. No other distant metastases were seen. A biopsy of the C1 mass was composed of sheets of epithelioid cells. Tumour cells were immunonegative for S100 and INI1 and exhibited strong keratin and nuclear brachyury staining (Fig. 1b, d, f and Supplementary Fig. 1). Based on these histopathological findings, a diagnosis of PDC was established. Similar to patient 1, patient 2 was treated with 4 cycles of vincristine, doxorubicin and cyclophosphamide with alternating ifosfamide and etoposide with reduction in size of the primary mass and pulmonary nodule. Proton beam therapy was declined and treatment discontinued based on the family’s wishes. Patient 2 developed locally recurrent disease with increased size of the pulmonary nodule, and was biopsied from the C1 mass for WGTA.

### Genomic and transcriptomic analysis of poorly differentiated paediatric chordomas

WGTA detected low somatic TMBs for both cases (1.83 and 1.75 mutations per megabase for patients 1 and 2, respectively). Notable alterations in both cases included single copy losses affecting *SMARCB1* (Fig. 1g), that were associated with low expression of *SMARCB1* compared to both The Cancer Genome Atlas (TCGA) sarcoma^18^ data set and a pan-cancer cohort of adult cancers, referred to as POG570, sequenced as part of the POG programme^19^ (Fig. 1h and Supplementary Table 1). In patient 1’s tumour, we detected apparent biallelic loss of *SMARCB1* as a result of an inverted translocation between *SMARCB1* intron 3 and a non-coding region on chromosome 5 (hg19 chr22:24138567–chr5:176754524). Consistent with diagnostic IHC testing, the *TBXT* gene that encodes the transcription factor brachyury was highly expressed in both samples compared to the TCGA sarcoma and POG570 data sets (Fig. 1h). WGBS data revealed hypomethylation of the *TBXT* promoter (−1800 to +3000 base pairs relative to the transcription start site), compatible with the notion that loss of *TBXT* promoter DNA methylation may drive increased gene expression in these chordomas (Fig. 1i).

### Immune markers associated with paediatric chordoma

**Fig. 2:**
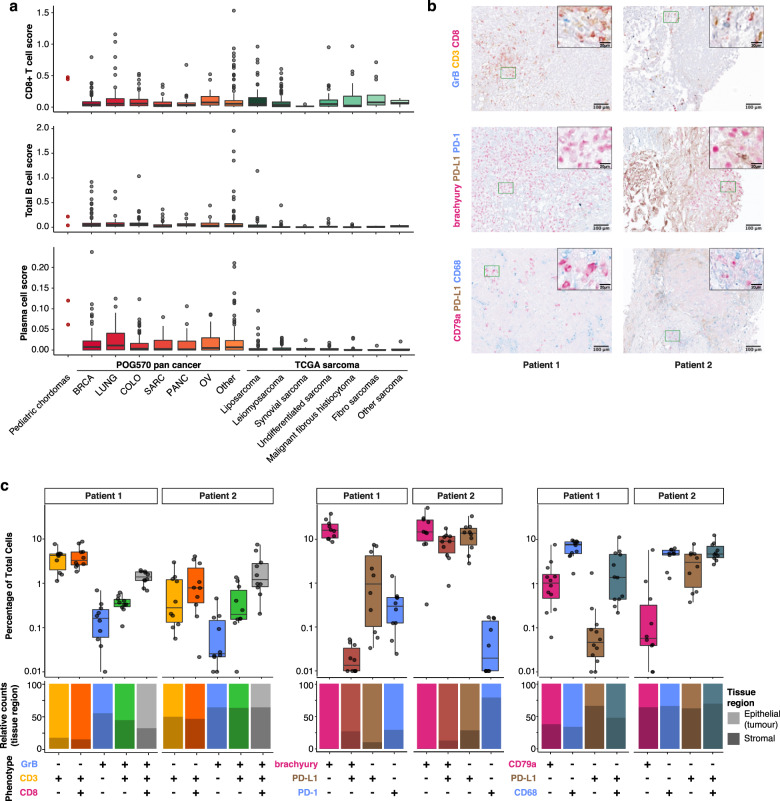
Immune microenvironment of poorly differentiated chordomas. **a** CIBERSORT estimation of CD8+ T cell, B cell and plasma cell abundance from bulk paediatric chordoma RNA sequencing data in paediatric chordomas, POG570 adult cancers^19^ and the TCGA sarcoma data set^18^. CD8+ T cell score (top panel) for patient 1 was 0.44 and for patient 2, 0.48 compared to the other cancer types, i.e. POG570 pan cancer adult solid tumours median score = 0.05 (BRCA, *n* = 144; LUNG, *n* = 67; COLO, *n* = 87; SARC, *n* = 47; PANC, *n* = 42; OV, *n* = 28; other, *n* = 155), TCGA sarcoma median score = 0.04 (*n* = 255). Similarly, B cell scores (middle panel) for patient 1 = 0.04, patient 2 = 0.22, POG570 median score = 0.04, TCGA sarcoma median score = 0.01. Plasma cell scores (bottom panel) for patient 1 = 0.06, patient 2 = 0.12, POG570 median score = 0.01, TCGA sarcoma median score = 0.00. **b** Example ×20 images of multiplex IHC staining with granzyme B (GrB)/CD3/CD8, brachyury/PD-L1/PD-1 and CD79a/PD-L1/CD68 panels. **c** Percentage of cells stained with the respective antibodies from paediatric chordoma patients 1 and 2. Individual points represent the respective cellular fraction for each population measured from independent images (*n* ≥ 10) taken from different locations of the slide for each patient sample. The relative proportion of either epithelium- or stroma-associated cells for each cellular population is displayed in the bottom panel. Box plots in **a**, **c** represent median, upper and lower quartiles, and whiskers represent limits of the distributions (1.5-times interquartile range).

Previous studies indicated linkages between SWI/SNF mutations and tumour immunogenicity^14,15,20^. We explored the immune microenvironment of poorly differentiated *SMARCB1*-deficient chordomas using CIBERSORT^21^ to estimate the relative abundance of immune cell types from RNA sequencing (RNA-seq) data. Gene expression-based estimates of immune cell abundance have the potential to identify patients that may respond to ICIs across multiple cancer types, including sarcomas^22,23^. Our analysis revealed that both chordomas exhibited high CD8+ T cell expression signatures compared to several other paediatric and adult cancer data sets, including paediatric cranial and extra-cranial RTs that are similarly *SMARCB1*/INI1-deficient^24,25^ (Fig. 2a and Supplementary Fig. 2). Plasma and B cell abundance estimates were similarly high in the paediatric chordomas, particularly for patient 2, indicating a heterogenous population of lymphocytes present in the tumour microenvironment (Fig. 2a).

To confirm and further explore the CIBERSORT-predicted high CD8+ T cell expression signatures, tumour biopsy samples were analysed using multiplex IHC staining. Staining with a T cell panel confirmed the presence of CD3+CD8− and CD3+CD8+ T cells, including granzyme B (GZMB)+CD3+CD8+ T cells in both samples (Fig. 2b, c). Gene expression analysis revealed high expression of *CD274* (programmed cell death ligand 1 (PD-L1)) compared to TCGA sarcoma (patient 1: 89th percentile, patient 2: 97th percentile) and POG570 pan-cancer data sets (patient 1: 83rd percentile, patient 2: 92nd percentile), indicating the presence of PD-L1-mediated immune evasion. In agreement with RNA expression data, IHC staining revealed PD-L1-positive cells in both tumour samples, with higher expression in patient 2 compared to patient 1. PD-L1 was expressed by both tumour and non-tumour cells as evidenced by the presence of PD-L1+/TBXT+ tumour cells in patient 2 and PD-L1+/CD68+ macrophages in patients 1 and 2 (Fig. 2c). Both samples also harboured CD79a+ B and plasma cells, which have been associated with ICI response in other cancers^26,27^.

### Clinical response to nivolumab

**Fig. 3:**
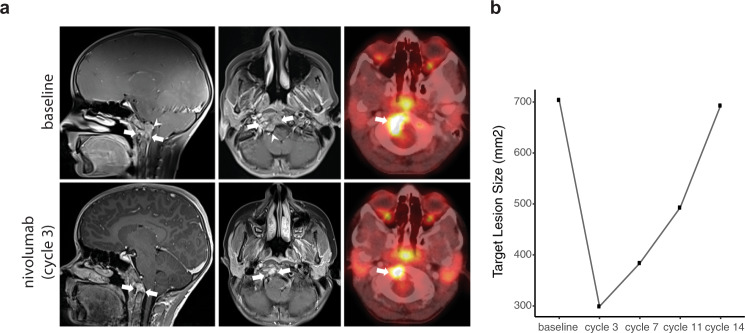
Response of a poorly differentiated paediatric chordoma to nivolumab. **a** Sagittal (left) and axial (middle) post-contrast MRI and concurrent PET/CT (right) images prior to nivolumab therapy (top panel) demonstrate an enhancing solid mass involving the clivus, basiocciput and right occipital condyle (arrows), with effacement of the premedullary cistern and indentation upon the brainstem (arrowheads) and a large focus of metabolic activity at the tumour site (SUVmax 8.2; arrow). Follow-up images (lower panels) 3 months after the initiation of nivolumab therapy show substantial decrease in size of the mass (arrows), with resolution of mass effect upon the brainstem. The concurrent PET/CT image shows attendant decrease in the region of metabolic activity (SUVmax 6.0; arrow), indicating a partial radiographic response. **b** Target lesion size at baseline and following 3, 7, 11 and 14 cycles of nivolumab.

While hypermutated tumours have been associated with response to ICIs^22,28,29^, paediatric cancers typically exhibit low TMB. Despite a low mutation burden as measured from whole-genome sequencing, estimates of CD8+ T cell abundance provided a rationale for ICI therapy^13,22^. Unfortunately, patient 1 died shortly after tumour sequencing and was not treated with immunotherapy or other molecularly targeted agents. The molecular tumour board recommended nivolumab, an anti-PD1 ICI with paediatric safety data, for patient 2 as a result of the evidence of a T cell-inflamed tumour. Given the lack of a suitable clinical trial, nivolumab was obtained via compassionate access following the failure of front line therapy. Following initiation of nivolumab, the patient experienced clinical improvement in symptoms, including decreased pain and improved mobility. MRI and PET/CT following three cycles of nivolumab showed partial response by Response Assessment in Neuro-Oncology (RANO) criteria, with an overall decrease in size (58% reduction in target lesion) and mass effect on the adjacent medulla (Fig. 3a, b). This partial response corresponded with a decrease in FDG-avidity (SUVmax 8.2 at baseline compared to 6.0 after 3 cycles). Although target lesion size remained smaller after 7 and 11 cycles compared to baseline, follow-up MRIs showed slow progression over time compared to documented best response. The patient remained on nivolumab for 14 cycles enabled by ongoing compassionate access to therapy, and continued to experience clinical benefit with an excellent functional status and quality of life.

### Brachyury elicits patient-derived T cell receptor (TCR) activation

**Fig. 4:**
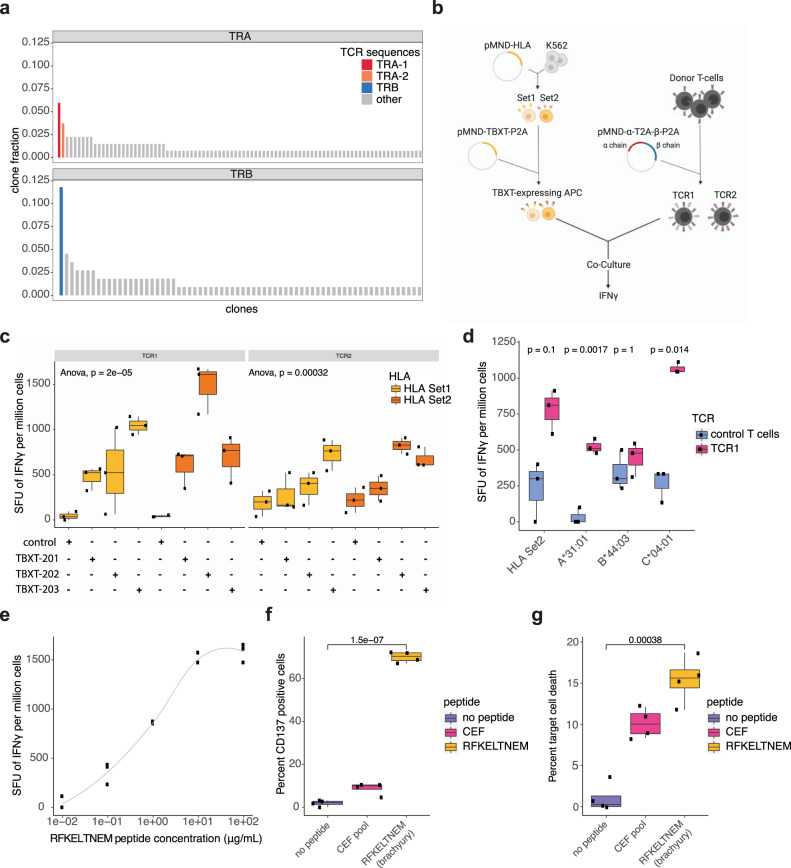
Patient-derived TCR activation and cell killing in response to a brachyury peptide antigen. **a** Unique TCR alpha (TRA) and TCR beta (TRB) chain sequences were inferred using bulk tumour RNA sequencing data from patient 2. The relative fraction of each sequence compared to all TRA and TRB aligned sequences is plotted on the *y*-axis. The two dominant TRA sequences TRA-1 and TRA-2 (red and orange bars, respectively) and the dominant TRB sequence (blue bar) were used for subsequent in vitro T cell activation ELISpot assays. **b** Schematic of ELISpot analysis of two reconstituted T cells (TCR1 and TCR2) expressing a patient-derived TRB chain and either of the two TRA chains, and synthetic antigen-presenting cells (APCs) transduced with one set of the patient’s classical HLA class I alleles (Set1 and Set2) and *TBXT*. IFNγ production is measured by the number of spot-forming units (SFU) per million cells. ELISpot results are shown for **c** TCR1 and TCR2 co-cultured with HLA Set1 or Set2 APCs expressing *TBXT-201*, *202*, *203* or control (*n* = 3 for each condition, one way ANOVA; *p* values: TCR1 *p* = 2e−05 and TCR2 *p* = 0.00032). **d** TCR1 or control T cells and either HLA Set2 or HLA-A*31:01, HLA-B*44:03 or HLA-C*04:01 alleles (*n* = 3 for all conditions, unequal variance, two-sided *T* test Bonferroni corrected; *p* values for HLA Set2 *p* = 0.1, HLA-A*31:01 *p* = 0.0017, HLA-B*44:03 *p* = 1 and HLA-C*04:01 *p* = 0.014); **e** varying concentration of brachyury peptide RFKELTNEM with HLA-C*04:01 APCs with TCR1 T cells. Percentage of **f** CD137-positive T cells and **g** target cell death following co-culture with no peptide, HLA class I control CEF peptide pool or the RFKELTNEM APCs and TCR1 T cells (*n* = 4 for all conditions, unequal variance, two-sided *T* test shows a significant difference between no peptide and RFKELTNEM in **f**, **g**). Box plots in **c**, **d**, **f**, **g** represent median, upper and lower quartiles, and whiskers represent limits of the distributions (1.5-times interquartile range).

Given the clinical and radiographic response to nivolumab, we surveyed the RNA-seq data from patient 2 for evidence of tumour-associated T cells that may have mediated an anti-tumour immune response. Extraction of TCR-containing reads using MiXCR^30^ revealed 134 TCR alpha chain (TRA) and 110 beta chain (TRB) aligned reads corresponding to 91 and 68 distinct clones, respectively (Supplementary Table 2). The most dominant TRB clone, CSASTGRGTEAFF, was supported by 11.8% of TRB aligned reads, while the two most dominant TRA clones were supported by 6.0% (TRA-1, CAVQSNYKLSF) and 3.7% (TRA-2, CALGAVYMDYVAGLYTDKLIF) of TRA aligned reads (Fig. 4a). To assess the relative dominance of the major TCR sequence identified in this sample, we compared the frequencies of the most dominant TRB clone from patient 2 to the POG570 cohort. The relative TRB dominance for patient 2 was >63% of POG570 dominant clones, indicating a relatively abundant TCR clone in the tumour microenvironment (Supplemental Fig. 2b). These findings derived from RNA-seq data indicate the presence of a predominant, clonally expanded T cell population in the tumour microenvironment.

Brachyury protein expression is normally restricted to the developing embryo but can be aberrantly expressed in tumours and elicit an immune response^31–33^. We speculated that the response of patient 2 to nivolumab may have been mediated by a brachyury tumour antigen. Peptide–major histocompatibility complex (MHC) (pMHC) binding predictions of brachyury with patient-derived HLA alleles identified 204 potential unique pMHCs for patient 2 (Supplementary Table 3). We therefore investigated the ability of patient 2 MHC class I-matched antigen-presenting cells (APCs) expressing brachyury to activate T cells harbouring the identified TCR sequences (TCR1 comprised of TRA-1 and TRB; TCR2 comprised of TRA-2 and TRB) (Fig. 4a, b). To facilitate experiments, patient 2 MHC class I molecules were split into two sets (HLA Set1 and Set2), and these sets were delivered to synthetic APCs along with three *TBXT* isoforms (see ‘Methods’). ELISpot analysis revealed responses of both TCR alpha–beta pairs (TCR1 and TCR2) to synthetic APCs expressing either set of patient-derived HLA alleles and *TBXT*, with the strongest response associated with TCR1 in co-culture with APCs expressing HLA Set2 and *TBXT-202* (Fig. 4c). Investigation of individual HLA alleles revealed the greatest response of TCR1 T cells in the presence of HLA-C*04:01 (Fig. 4d). To identify specific brachyury epitopes that mediated T cell stimulation in our model system, we screened peptides that were predicted to bind HLA-C*04:01 (Supplementary Table 3). We identified a peptide, RFKELTNEM, that induced T cell stimulation as measured by CD137 and interferon γ (IFNγ) expression, as well as elicited T cell-mediated target cell killing (Fig. 4e–g and Supplementary Fig. 3d, e). Together, our data indicate patient 2’s PDC tumour sample harboured a brachyury-specific CD8+ T cell clone. The presence of cytotoxic T cells and evidence for brachyury tumour antigen-mediated immune activity, along with high expression of PD-L1, were compatible with the notion that the observed responses to nivolumab may have been driven by disinhibition of this brachyury-specific T cell clone and potentially other tumour-reactive T cells.

### Methylation profiles reveal common immune pathway activities between chordoma and a subset of RTs

**Fig. 5:**
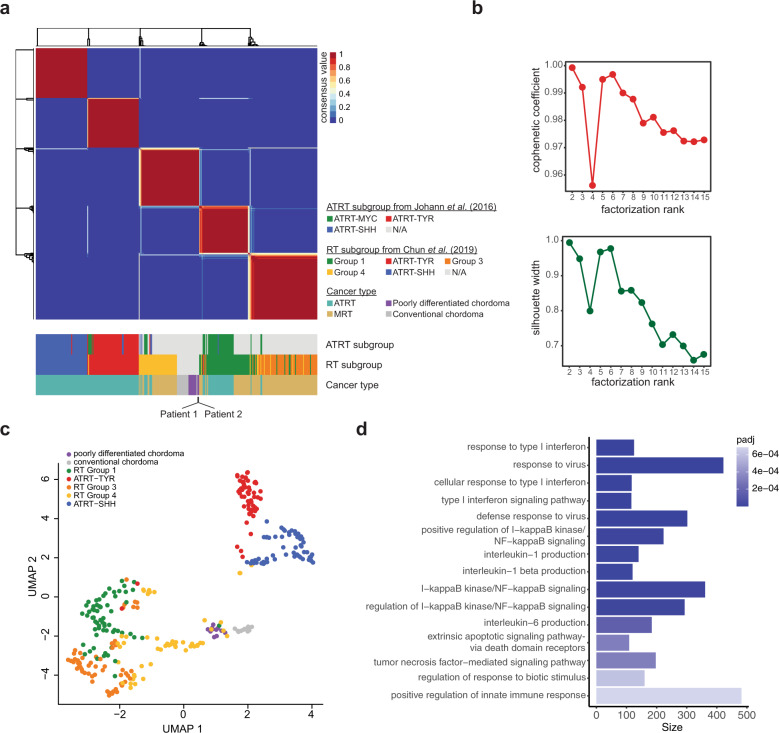
Methylation clustering reveals common immune response pathway activities with a subset of extra-cranial malignant rhabdoid tumours. **a** DNA methylation clustering using non-negative matrix factorization (NMF) of paediatric chordoma data from this study (*n* = 2) and Hasselblatt et al.^2^ (*n* = 23), atypical teratoid rhabdoid tumours (ATRT, *n* = 161) and extra-cranial rhabdoid tumours (MRT, *n* = 131). The respective ATRT subgroups identified in Johann et al.^74^ and rhabdoid tumour (RT) subgroups identified in Chun et al^17^. are displayed in the track below. **b** Cophenetic coefficients (top) and silhouette widths (bottom) for NMF cluster solutions from *k* = 2 to *k* = 15. A robust clustering result was obtained at *k* = 5, with high cophenetic coefficient and silhouette width. **c** Uniform manifold approximation and projection (UMAP) clustering of RTs with chordomas. **d** Gene set enrichment analysis of genes with differentially methylated CpGs in paediatric chordoma compared to RT subgroups 1–3 and 5 using a logistic regression model corrected for the number of CpGs in the gene set using methylGSA^73^. The number of genes associated with the gene ontology (GO) terms are shown and bars are coloured by FDR adjusted *p* values. The most significantly enriched terms, i.e. type I interferon, response to virus and type I interferon signalling pathway, had adjusted *p* values of 2.93e−08, 7.55e−08 and 1.13e−07, respectively.

Preliminary reports from phaseI/II clinical trials in solid paediatric tumours have indicated a generally low rate of response to ICIs^7–9^. However, ICI responses documented in individual patients participating in clinical trials, or receiving off-label therapy, has pointed to a potentially sensitive group of SWI/SNF-mutant paediatric tumours^9–11,13^. For example, increased sensitivities to T-cell mediated killing and improved survival after ICI treatments observed in clear cell renal cell carcinomas have been linked to SWI/SNF mutations^15,20^. In particular, applications of immunotherapies such as CAR-T cell and ICI treatments in paediatric and adult INI1-deficient RTs have been described^9,10,13,34–36^. RTs are characterized by heterogeneous immune phenotypes, with a subset of tumours exhibiting immunologically hot phenotypes^14,17^. Given the common loss of INI1 in RT and PDC, and prevalence of CD8+ T cell infiltration in a subset of RTs and the two chordomas presented here, we sought to determine if paediatric chordomas were more similar to immunologically hot RTs than to immunologically cold RTs. DNA methylation clustering of chordoma and extra-cranial malignant RT (MRT) and cranial atypical teratoid RT (ATRT) cases using non-negative matrix factorization (NMF; Fig. 5a, b) and uniform manifold approximation and projection (UMAP) algorithms (Fig. 5c) consistently showed that chordoma cases clustered most closely with RT subgroup 4 characterized by higher infiltration levels of cytotoxic CD8+ T cells compared to the immunologically cold ATRT-SHH and -TYR subgroups. While hierarchical clustering of conventional chordoma and PDCs was consistent with previous observations that these are distinct molecular subgroups^2^ (Supplementary Fig. 4), both chordoma subgroups were similarly correlated with RT subgroup 4. RT subgroup 4 was largely comprised of extra-renal MRTs and characterized by gene expression and DNA methylation profiles that suggested increased immune response pathway activities and tumour-infiltrating T cells compared to other RT subgroups^17^. To explore molecular distinctions between chordomas and RT subgroups, we compared methylation profiles in chordomas to RT subgroups 1–3 and 5 (i.e. excluding subgroup 4). We observed enrichment of genes within differentially methylated regions that were involved in type I interferon response and response to viral infection pathways when chordomas were compared to RT subgroups 1–3 and 5 (Fig. 5d and Supplementary Table 4). These results are compatible with the notion that epigenetic dysregulation of immune pathways and increased CD8+ T cell infiltration may be a feature shared between chordoma and a subset of RTs.

## Discussion

Diverse immune profiles across paediatric solid tumour patients have been described^37^, and there is speculation that immunologically hot tumours may be particularly sensitive to ICI therapy as has been proposed for adult solid tumours^22,38–41^. Interim results from prospective trials have indicated limited responses to ICIs in paediatric solid tumours, and argued that PD-L1 expression on its own is likely insufficient to predict response across paediatric cancers^7,9^. Some paediatric patients with INI1-negative tumours have demonstrated clinical benefit from ICIs, including paediatric chordoma and RTs^9,10,13^. The beneficial response to the ICI, nivolumab, in a paediatric chordoma we describe here support these initial observations, and add further support for antigen-directed T cell responses and cytotoxic T cell infiltration that contribute to establishing an inflamed tumour microenvironment. Thus, our observations provide evidence that an immunologically hot phenotype is a compelling marker correlated with ICI response, and may provide mechanistic insight into the mediators of this response. Epigenomic analyses points to common immune related pathway activities in immunologically hot RTs and chordomas, indicating a shared immune phenotype that could potentially be exploited with immune targeting therapies as demonstrated for patient 2. The collective evidence from past studies and our observations presented here indicate that profiling of immune phenotypes as part of prospective clinical trials will be important to evaluate whether immune infiltrates are predictive of ICI response in chordoma, RTs and paediatric malignancies more broadly.

Tumour antigens are potentially targetable mediators of anti-tumour immune responses^42,43^, particularly in tumours with low TMB. A previous HLA-A*02:01 restricted brachyury epitope (WLLPGTSTL)^31^ has been shown to stimulate T cell-mediated cell killing, and brachyury-targeting T cells have been reported in chordoma patients including adult patients who have responded to immunotherapy^44^. Given the absence of a single clonal nonsynonymous mutation in patient 2, aberrant expression of the brachyury tumour antigen was a strong candidate target for the anti-tumour response following treatment with nivolumab in patient 2. Our discovery of brachyury-targeting tumour-infiltrating lymphocytes in chordomas, along with expression of PD-L1 supports further investigation of similar patients with immune modulating therapies^44–47^.

## Methods

### Ethics and consent

This work was approved and conducted under the University of British Columbia Children’s and Women’s Research Ethics Board (H13–01640). All patients presented in this publication were approached for enrolment by a study trained oncologist and provided written informed consent.

### Diagnostic biopsy preparation and staining/IHC

Needle core biopsies of tumour were fixed in 10% neutral buffered formalin for a minimum of 8 h prior to automated tissue processing and paraffin embedding. Four micron formalin-fixed paraffin embedded tissue sections were cut by microtome and mounted onto charged glass slides. Tissue sections were either subjected to automated routine staining with haematoxylin and eosin, or subjected to IHC using the Ventana Benchmark XT automated stainer. Antibody staining conditions were as follows: anti-pan-keratin (clone AE1/AE3, MS-343-R7 Thermo Scientific, ready to use) incubation at 37 °C for 32 min, anti-BAF47/INI1 (clone 25, 612110 BD Bioscience, 1:100) incubation at 37 °C 32 min and anti-S100 (clone 4C4.9, 790–2914 Ventana, prediluted) incubation at room temperature 4 min. For brachyury IHC (EPR18113, Abcam, 1:2000), staining was performed at PhenoPath Laboratories (Seattle, WA).

### PCR-free DNA library preparation

Nucleic acid was extracted from fresh frozen using RLT buffer (Qiagen). Constitutional DNA representing normal cells was extracted from peripheral blood. To minimize library bias and coverage gaps associated with PCR amplification of high GC or AT-rich regions, a version of the TruSeq DNA PCR-free kit (E6875–6877B-GSC, New England Biolabs), automated on a Microlab NIMBUS liquid handling robot (Hamilton) was employed. Briefly, 500 ng of genomic DNA was arrayed into wells in a 96-well microtitre plate and subjected to shearing by sonication (Covaris LE220). Sheared DNA was end-repaired and size-selected using paramagnetic PCRClean DX beads (C1003–450, Aline Biosciences), targeting a 350–450 bp size range. After 3’ A-tailing, full length TruSeq adapters were ligated to DNA fragments. Libraries were purified using paramagnetic beads (Aline Biosciences). Prior to sequencing, PCR-free genome library concentrations were quantified using a qPCR Library Quantification kit (KAPA, KK4824).

### Bisulfite library construction

To track the efficiency of bisulfite conversion, 10 ng lambda DNA (Promega) was spiked into 1 µg genomic DNA quantified using Qubit fluorometry and arrayed in a 96-well microtitre plate. DNA was sheared to a target size of 300 bp using Covaris sonication and the fragments were subject to end-repair and phosphorylation in a single reaction using an enzyme mixture (New England Biolabs) containing T4 DNA polymerase, Klenow DNA Polymerase and T4 polynucleotide kinase, and incubated at 20 °C for 30 min. Repaired DNA was purified in 96-well format using PCR Clean DX beads (Aline Biosciences, USA), and 3’ A-tailed (adenylation) using Klenow fragment (3’ to 5’ exo minus) and incubation at 37 °C for 30 min prior to enzyme heat inactivation. Cytosine methylated paired-end adapters (5’-AmCAmCTmCTTTmCmCmCTAmCAmCGAmCGmCTmCTTmCmCGATmCT-3’ and 3’-GAGmCmCGTAAGGAmCGAmCTTGGmCGAGAAGGmCTAG-5’) were ligated to the DNA at 20 °C for 15 min and adapter flanked DNA fragments bead purified. Bisulfite conversion of the methylated adapter-ligated DNA fragments was achieved using the EZ Methylation-Gold kit (Zymo Research) following the manufacturer’s protocol. Five cycles of PCR using HiFi polymerase (Kapa Biosystems) was used to enrich the bisulfite converted DNA. Post-PCR purification and size-selection of bisulfite converted DNA was performed using 1:1 PCR Clean DX beads. To determine final library concentrations, fragment sizes were assessed using DNA1000 assay (Agilent) and DNA quantified by Qubit fluorometry.

### Strand-specific RNA-seq library preparation

Qualities of total RNA samples were determined using an Agilent Bioanalyzer RNA Nanochip or Caliper RNA assay and arrayed into a 96-well plate (Thermo Fisher Scientific). Polyadenylated (poly(A)) RNA was purified using the NEBNext Poly(A) messenger RNA (mRNA) Magnetic Isolation Module (E7490L, NEB) from 350 ng (patient 1) and 192 ng (patient 2) total RNA normalized in 35 µL for DNase I treatment (1 Unit, Invitrogen). DNase-treated RNA was purified using RNA MagClean DX beads (Aline Biosciences, C-1005-250) on a Microlab NIMBUS liquid handler (Hamilton Robotics, USA). mRNA selection was performed using NEBNext Oligod(T)25 beads (NEB). First-strand cDNA was synthesized from the purified polyadenylated mRNA or rRNA depleted total RNA using the Maxima H Minus First Strand cDNA Synthesis kit (Thermo-Fisher, FSSP9760210) and random hexamer primers at a concentration of 5 µM along with a final concentration of 1 µg/µL Actinomycin D, followed by PCRClean DX bead purification on a Microlab NIMBUS robot (Hamilton Robotics, USA). Second strand cDNA was synthesized following the NEBNext Ultra Directional Second Strand cDNA Synthesis protocol (NEB) which incorporates dUTP in the dNTP mix, allowing the second strand to be digested using USERTM enzyme (NEB) in the post-adapter ligation reaction, thus achieving strand specificity. cDNA was fragmented using Covaris LE220 sonication to achieve 200–250 bp average fragment lengths. The paired-end sequencing library was prepared using a strand-specific, plate-based library construction protocol on a Microlab NIMBUS robot (Hamilton Robotics, USA). The sheared cDNA was subject to end-repair and phosphorylation in a single reaction using an enzyme premix (NEB) containing T4 DNA polymerase, Klenow DNA Polymerase and T4 polynucleotide kinase. End-repaired cDNA was purified in 96- well format using PCRClean DX beads, and 3’ A-tailed (adenylation) using Klenow fragment (3’ to 5’ exo minus). Illumina PE adapters were ligated and adapter-ligated products were purified using PCR Clean DX beads, then digested with USERTM enzyme (1 U/µL, NEB) followed by 13 cycles of indexed PCR using Phusion DNA Polymerase (Thermo Fisher Scientific Inc. USA) and Illumina’s PE primer set. The PCR products were purified twice and size-selected using a 1:1 PCRClean DX beads-to-sample ratio. The eluted DNA quality was assessed using the Caliper LabChip GX for DNA samples and the High Sensitivity Assay (PerkinElmer, Inc. USA). Quantification was performed using a Quant-iT dsDNA High Sensitivity Assay Kit on a Qubit fluorometer (Invitrogen). Libraries were then pooled and size-selected to adjust the final library molar concentration for sequencing.

### Sequencing

Tumour genomes were sequenced on HiSeqX using v2.5 chemistry and paired-end 150 base reads. Bisulfite paired-end libraries were sequenced on the HiSeqX to a length of 150 base reads. Transcriptomes were sequenced on NextSeq500 using v2 chemistry to a length of 75 bp. Sequencing coverage data are listed in Supplementary Table 5.

### DNA methylation analysis from WGBS

Bisulfite genomes were aligned to the human reference genome (hg19) using novoAlign (v.3.04.06) (http://www.novocraft.com/products/novoalign/) and fractional methylation was assessed using in-house novo5mC (0.8.9d) (https://svn.bcgsc.ca/bitbucket/projects/EDCC/repos/opencemt/browse/WGBS/QC).

### Gene expression

RNA-seq reads were aligned using STAR (v.2.5.2b) and expression was quantified using RSEM (v.1.3.0) as TPM. Publicly available TCGA sarcoma RNA-seq samples in Xena Public Data Hubs (https://xena.ucsc.edu/public). STAR and RSEM were generated from the hg38 reference genome (http://hgdownload.cse.ucsc.edu/goldenPath/hg38/bigZips/) and gene annotations were based on EnsEMBL v.85^48^. Samples used for gene expression analysis are listed in Supplementary Table 1.

### Somatic mutation detection

Sequence reads from normal and tumour whole genome libraries were aligned to the human reference genome (hg19) using the Burrows–Wheeler Alignment tool^49^ (v0.7.6a). Tumour genome sequences were compared to those from the patient’s constitutional (normal) DNA to identify somatic alterations. Regions of copy number variation and losses of heterozygosity were identified using the Hidden Markov model-based approaches CNAseq2 (v0.0.6)^50^ and APOLLOH (v0.1.1)^51^, respectively. Somatic single nucleotide variants (SNVs) were identified using two approaches: (1) putative somatic variant calls from SAMtools^52^ (v0.1.17) with subsequent scoring by machine-learning based MutationSeq^53^ (v4.3.5), and (2) identification and scoring with the joint caller Strelka^54^ (v1.0.6). Small (<20 bp) insertions and deletions (indels) were identified using Strelka with QSI ≥ 15. Whole-genome TMB was defined as the sum of all somatic single nucleotide variants and indels that were marked as passed by Strelka, divided by the genome size (2864785220 bases). Structural variants (SVs) in RNA-seq data were identified using the assembly-based tools ABySS v1.3.4^55^ and TransABySS (v1.4.10)^56^ and alignment-based tools Chimerascan^57^ (v0.4.5) and DeFuse^58^ (v0.6.2); SVs in the DNA sequence data were identified using assembly-based tools ABySS and Trans ABySS and alignment-based tools Manta^59^ v1.0.0 and Delly^60^ v0.7.3. Putative SV calls identified from the DNA and RNA sequences were merged into a consensus caller MAVIS^61^ (v2.1.1), where they were clustered, computationally validated and annotated against constitutional DNA to provide somatic and germline structural variant calls. Both DNA- and RNA-derived structural variant calls were additionally filtered to identify those called by more than one tool, and for which a contig could be assembled that aligned across a candidate genomic breakpoint. DNA SV calls were further filtered to exclude events with identical genomic breakpoints in multiple samples, removing potentially confounding germline variants and technical artefacts. Variants were annotated to genes using SNPEff (v3.2)^62^ with the EnsEMBL database (v69). TCR alpha and beta clones were detected from RNA-seq data using MiXCR (v3.0.5)^30^. POG570 solid tumour samples that were not obtained from lymph node, bone marrow or peripheral blood and had >1 unique TRB sequence detected were included in Supplementary Fig. 2b (*n* = 447).

### Immune cell deconvolution

RNA-seq reads from paediatric chordomas (*n* = 2), the adult POG570 pan-cancer cohort (*n* = 570), TARGET RTs (*n* = 66), TARGET clear cell sarcoma of the kidney (*n* = 13), TARGET osteosarcoma (*n* = 87) and TCGA sarcoma data set (*n* = 255) were aligned and processed using JAGuaR (v2.0.3)^63^ as described in Jones and colleagues (2010)^50^. Gene level RPKM (reads per kilobase per million mapped reads) were calculated on the basis of EnsEMBL gene models (v69). Immune cell type deconvolution was performed with the CIBERSORT R package (v1.04)^21^, using the LM22 cell subtype signature in absolute mode, with 1000 permutations and no quantile normalization as described in Pender and colleagues^22^. A list of samples used for this analysis is supplied in Supplementary Table 1.

Raw RNA counts from 18 tumour samples with diverse diagnoses (undifferentiated sarcoma, atypical teratoid RT, Wilms tumour, neuroblastoma, acute lymphoblastic leukaemia, hepatoblastoma, nasopharyngeal carcinoma, rhabdomyosarcoma and Ewing’s sarcoma) sequenced as part of the Treehouse Initiative were converted to RPKM on the basis of Gencode v23 model (https://www.gencodegenes.org/) and similarly analysed using the CIBERSORT R package.

### Multiplex IHC

Slides containing tissue sections from the same tissue blocks that were processed for sequencing were baked, deparaffinized and antigens were retrieved using Diva decloaker (Biocare). Endogenous peroxidase was blocked using peroxidized-1 followed by blocking with Background Sniper (Biocare) and staining with anti-granzyme B (GrB-7, MA1-35461 Thermo Fisher, 1:20), CD8 (C8/144B, 108M-94 Cell Marque, 1:250), CD3 (Sp7, M3074 Spring Bioscience, 1:500), PD-L1 (SP142, M4422 Spring Bioscience, 1:100), PD-1 (NAT105, 315M-94 Cell Marque, 1:100), brachyury/TBXT (EPR18113, ab209665 Abcam, 1:1500), CD79a (SP18, M3182 Spring Bioscience, 1:600), CD68 (KP-1, CM033 Biocare, 1:100) and chromogens 3, 3’ Diaminobenzidine (BC-IPK5010G80, Biocare) or High Def Yellow (ADI-950-170-0030, Enzo), Ferengi Blue (BC-IPK5027G20) and Warp red (BC-IPK5024G80). Slides were counterstained with haematoxylin. Slides were scanned on a Pannoramic Midi scanner (3DHistech Ltd.) and analysed using the InForm and Vectra image analysis software (Perkin Elmer). Ten-to-12 images per slide taken from the same sample were analysed for each multiplex panel. Percentage of positive cells as determined by five independent algorithms were averaged for each image.

### Neoantigen prediction

TBXT protein sequence was downloaded from Uniprot (J3KP65-1). Class I HLA genotypes for each patient were derived using OptiType^64^ run separately on the tumour WGS, tumour RNA-seq, and normal WGS data sets. NetMHCpan 4.0^65^ was used to predict pMHC binding for 8–11mer peptides from TBXT. NetMHCpan was run in Eluted Ligand mode, which predicts the likelihood that a peptide–MHC would be observed on the surface of a cell. We selected peptide–MHC combinations with Rank ≤2% as predicted binders.

### Design of *TCR*, *HLA* and *TBXT* sequences

Recombinant TCRs were designed based on the RNA-seq data from patient 2 using Geneious (v.8.1.4) and the IMGT/GENE Database^66^. To minimize mismatch pairing with the endogenous TCRs, mouse constant regions were used for both the alpha and beta chains as previously described^67^. Recombinant HLAs were designed based on the patient 2 derived HLA-class I alleles and the sequences were retrieved from the EMBL-EBI Immune Polymorphism Database^68^. HLA-class I A, B and C alleles were split into two sets to facilitate testing (HLA Set-1: A*23:01, B*40:01, C*03:04; HLA Set-2 (A*31:01, B*44:03, C*04:01). The *TBXT* (ENSG00000164458) isoform *TBXT-201* and *TBXT-202* cDNA reference sequences were retrieved from the NCBI Consensus Coding Sequence Database (CCDS ID: CCDS5290 and CCDS59045, respectively). The nucleotide sequence for *TBXT-203* was retrieved from NCBI (Gene ID: NM_001366285.1)

### Molecular cloning, lentiviral manufacture and titration

The *TCR*, *HLA*, and the *TBXT* sequences were separately cloned into pMND lentivirus vectors. For recombinant TCRs, the patient-derived *TCR* alpha and beta chains were synthesized de novo and cloned into lentiviral transfer plasmid as a biscistronic alpha-beta gene cassette containing a downstream mStrawberry reporter gene, giving two recombinant TCRs: TCR1 (TRA-1 CAVQSNYKLSF; and TRB CSASTGRGTEAFF) and TCR2 (TRA-2 CALGAVYMDYVAGLYTDKLIF; and TRB CSASTGRGTEAFF). The *TBXT-201*, *TBXT-202* and *TBXT-203* sequences were synthesized de novo and cloned into lentiviral transfer plasmids containing a downstream mStrawberry reporter gene. The *HLA* sequences were synthesized de novo and cloned into lentiviral transfer plasmids.

To generate each lentivirus, 80 µg of the specific transfer plasmid was incubated for 30 min at room temperature, with 72 µg of the pCMV-ΔR8.91plasmid, 8 µg of the pCMV-VSV-G plasmid, and 430 µL of TransIT-LT1 reagent (Mirus, MIR2305) in a mix made up to 8 mL with OptiMEM (Gibco, 31985062). HEK 293 T/17 cells (ATCC® CRL-11268^TM^) were transfected. Viral supernatants were collected and virus resuspended in OptiMEM.

Titration and sorting of the transduced K562-APCs was done by staining the cells with HLA-A-PECY5 (Creative Diagnostic, DCABH-4529 1:50), HLA-B-APC (Creative Diagnostic, CABT-BL7610, 1:50) and HLA-C-PE (BD, 566372, 1:50) surface staining antibodies, along with the mStrawberry reporter transgene expressed with the recombinant *TBXT* transgenes. Representative images demonstrating the efficacy of lentiviral transduction and multiplicity of infection for *TCR1*, *TBXT-201* and HLA-C*04:01 constructs are shown in Supplementary Fig. 3a–c. Negative controls included untransduced K562 cells. Cells and antibodies were incubated at 4 °C for 30 min, then washed by adding 2 mL of FACS media (1× D-PBS 14190-144, Gibco with 2% HI-FBS 10082-147, Gibco) to each tube and centrifuging for 10 min at 400 × *g* at 4 °C, discarding the supernatant before resuspension in 400 μL of cold FACS media. The titration of TCR1 and TCR2 over K562 cells or the sorting of TCR1 and TCR2 transduced CD8+ T cells involved washing the cells after transduction by adding 2 mL of FACS media (1× D-PBS 14190-144, Gibco with 2% HI-FBS 10082-147, Gibco) to each tube and centrifuging for 10 min at 400x g at 4 °C, discarding the supernatant before repeating the wash and resuspending the cells in 400 μL of cold FACS media.

Acquisition of titration data was performed on the BD FACSymphony cell analyser, and analysed using the FlowJo and GraphPad Prism software version 8.0.0 for windows (GraphPad Software, California, USA). For cell sorting, the BD FACSAria Fusion was used along with the FACSDiva software. FACS analysis involved capturing the data on FSC-A, FSC-H, FSC-W, SSC-A, SSC-H, SSC-W, 561 nm laser with the 670/14 filter (PECY5-HLA-A), the 640 nm laser with the 670/30 filter (HLA-B - APC), 560 nm laser with the 582/15 filter (PE-HLA-C), and 560 nm laser with the 640/20 filter (mStrawberry reporter transgene).

### Viral transduction and cell line generation

Two separate K562 (ATCC, CCL-243) cell lines were developed expressing three of the patient’s six HLA class-I alleles: HLA-ABC Set1 and Set2 transduced with the *TBXT* lentivirus. Donor CD8+ T cells were isolated from a source of peripheral blood mononuclear cells (PBMCs) (Human Peripheral Blood Leukopak, Stemcell Technologies) using the Miltenyi MACS human CD8+ isolation kit, as per the manufacturer’s protocol (Miltenyi, 130-096-495) and using an in-house MACS separation buffer. The CD8+ T cells were resuspended in RPMI-1640 supplemented media with 300 U/mL rhIL-2 (Peprotech, 200-02 and StemCell, 78036.3), 100 ng/mL of anti-CD3 (clone OKT3, eBioscience 16-0037-85) and 100 ng/mL anti-CD28 (clone CD28.2, BioLegend 302943) soluble antibodies. CD8+ T cells were transduced with lentivirus carrying *TCR1* and *TCR2* sequences and sorted for mStrawberry expression via flow cytometry (BD Aria and BD FACSDiva software). *TCR1* and *TCR2* transduced CD8+ T cells were expanded in RPMI-1640 supplemented media with 300 U/mL rhIL-2 (Peprotech, 200-02 and StemCell, 78036.3) and allogenic irradiated feeder cells at a ratio of 1 TCR T cell to 200 irradiated feeder cells.

### ELISpots

ELISpot plates were coated with IFNγ capture antibody (2 μg/mL per well, Mabtech mAb 1-D1K, 3420-7) in D-PBS and then blocked with RPMI-1640 supplemented media. For experiments in Fig. 4c, d, APCs co-expressing HLA Set1, Set2 or individual alleles from Set 2 (HLA-A*31:01, HLA-B*44:03 or HLA-C*04:01) with *TBXT* or negative control were co-cultured with TCR1 or TCR2 T cells at an effector to target (E:T) ratio of 1:1 was followed by detection of IFNγ with anti-IFNγ biotinylated antibody (1 µg/mL per well; Mabtech, mAb 7-B6-1, 3420-9H), streptavidin-HRP (diluted 1:100 with D-PBS, Mabtech, 3310-9-1000), and 3'3'5'5’-tetramethylbenzidine (TMB substrate) (Mabtech, 3652-F10). The ELISpot plate was imaged on AID automated microplate ELISpot reader and associated software (AID ELISpot Version 7.0). For experiments in Fig. 4e, 5 × 10^4^ (per reaction) APCs expressing the recombinant HLA-A*04:01 allele, were peptide pulsed decreasing concentrations starting at 100 µg/mL and decreasing 10 fold until APCs were pulsed with 0.01 µg/mL of the RFKELTNEM peptide (GenScript). The peptide pulsed APCs were co-cultured with TCR1 T cells at an E:T ratio of 1:1, followed by IFNγ detection as previously described. Figure 4b was created with BioRender.com.

### CD137 activated expression and cytotoxic assays

For Fig. 4f, 5 × 10^4^ (per reaction) APCs expressing the recombinant HLA-C*04:01 allele, were peptide pulsed with 10 µg/mL of the RFKELTNEM peptide (GenScript) or were left not peptide pulsed. For the CEF control, HLA Set2 APCs were peptide pulsed with 1 µg/mL of the CEF peptide. These APCs were then co-cultured with TCR1 T cells at an E:T ratio of 4:1 for 24 h before being stained with the CD8 AF-700 (clone SK1, 344724 BioLegend, 1:100) CD3-efluor 450 (clone 17A2, 48-0032-82, eBioscience, 1:200), CD137-AF647 (clone 4B4-1, A51019 ThermoFisher, 1:50) conjugated flow antibodies. Cells and antibodies were incubated at 4 °C for 30 min, then washed by adding 2 mL of FACS media (1x D-PBS 14190-144, Gibco with 2% HI-FBS 10082-147, Gibco) to each tube and centrifuging for 10 min at 400x g at 4 °C, discarding the supernatant before resuspension in 400 μL of cold FACS media. Acquisition was performed on the BD FACSymphony cell analyser, and analysed using FlowJo and GraphPad Prism software version 8.0.0 for windows (GraphPad Software, CA, USA). FACS analysis of the samples involved capturing the data on FSC-A, FSC-H, FSC-W, SSC-A, SSC-H, SSC-W, 637 nm laser with the 670/30 (APC-CD137 antibody) and 710/50 (AF-700-CD8) filters, 405 nm laser with the 450/50 (eFluor 450-CD3), and the 561 nm laser with the 610/20 (RFP) filter. A figure illustrating an example of this strategy is shown in Supplementary Fig. 3d.

For Fig. 4g, 5 × 10^4^ (per reaction) APCs expressing the recombinant HLA-C*04:01 allele, were peptide pulsed with 10 µg/mL of the RFKELTNEM peptide (GenScript) or were left not peptide pulsed. For the CEF control, HLASet2 APCs were peptide pulsed with 1 µg/mL of the CEF peptide. These APCs were then co-cultured with TCR1 T cells at an E:T ratio of 4:1 for 4 h before being stained with the CD3-efluor 450 (clone 17A2, 48-0032-82, eBioscience, 1:100), HLA-A2-FITC (clone BB7.2, 343304 Biolegend, 1:200) conjugated flow antibodies, and the Fixable Viability Dye eFluor 780 (eBioscience 65-0865-14, 1:1000). Cells were stained with the antibodies and viability dye and incubated at 4 °C for 30 min, then the cells were fixed by adding 0.5 mL of fixation buffer (420801, Biolegend) and incubating the cells in the dark for 20 min, at 21 °C. The cells were then washed by adding 2 mL of FACS media to each tube and centrifuging for 10 min at 400 × *g* at 4 °C, discarding the supernatant before resuspension in 400 μL of cold FACS media. Acquisition was performed on the BD FACSymphony cell analyser, and analysed using FlowJo and GraphPad Prism software version 8.0.0 for windows (GraphPad Software, California, USA). FACS analysis of the samples involved capturing the data on FSC-A, FSC-H, FSC-W, SSC-A, SSC-H, SSC-W, 405 nm laser with the 450/50 (eFluor 450-CD3), the 488 nm laser with the 515/20 filter (HLA-A2-FITC), and 637 nm laser with the 780/60 (Fixable Viability Dye eFluor 780). A figure illustrating an example of this strategy is shown in Supplementary Fig. 3e.

### DNA methylation analysis

IDAT files from Illumina 450k methylation arrays, published in Hasselblatt et al.^2^, were analysed using the minfi R package (v1.32.0). Raw data were normalized, and probes that exhibited a *p* value of >0.01, aligned to common SNPs, or were previously reported to be cross reactive^69^ were removed from the analyses.

Fractional methylation values from WGBS were filtered to include CpG sites that were commonly profiled by the Infinium HumanMethylation450 bead chip array.

Unsupervised hierarchical clustering of paediatric chordoma fractional methylation was performed using the hclust and dist functions from the stats R package (v3.6.3) using 10,000 most variably methylated CpGs ranked by standard deviation. To visualize clustering results, a heatmap was generated using the heatmap3 R package (v1.1.7).

To reveal the extent of molecular similarities between RTs and *SMARCB1-*deficient chordomas, we analysed WGBS data generated from the POG chordoma cases (*n* = 2), and DNA methylation array data from chordoma cases published in Hasselblatt et al.^2^ (2016; *n* = 23 cases), RT cases from Chun et al.^17^ (2019; *n* = 161 cases from cranial atypical teratoid RTs (ATRTs) and 131 cases from extra-cranial malignant RTs (MRTs)). We first selected CpG sites that were commonly profiled using WGBS, Illumina Infinium 450 K HumanMethylation and 850 K EPIC array platforms. Next, we analysed CpG sites that were considered to be positively methylated by first removing CpG sites with 0% methylation across all 317 samples, and then selecting CpG sites with beta-values greater than 0.3 in at least 10% of samples^70,71^. These filtering steps yielded 248,121 CpG sites, which were then ranked using standard deviation to select the most variably methylated CpG sites. To reveal underlying clustering structures of the DNA methylation data, we performed an unsupervised NMF analysis using the top 10,000 most variably methylated CpG sites with a default Brunet algorithm, and 50 and 500 iterations for the rank survey and the clustering runs, respectively. The NMF method was implemented in the NMF R package (v0.20.2)^72^. The corresponding sample and subgroup information was visualized using the image function in R (v3.6.0). Unsupervised clustering using the UMAP algorithm was also performed on the 10,000 most variably methylated CpG sites using the umap R package (v0.2.6.0). The UMAP analysis was performed using 2,000 iterations during layout optimization (n_epochs), the minimum distance of 0.15 between points (min_dist) for better visualization of the global manifold structure of our data, and 20 nearest neighbours (n_neighbors) to allow 3–10% of the data set as the neighbours. For all other UMAP parameters, default settings were used.

### Pathway enrichment

Fractional methylation of CpG sites that were common among paediatric chordoma samples from this study, and Hasselblatt et al.^2^ were compared to cranial and extra-cranial malignant RT methylation subgroups 1, 2, 3 and 5. Significant differentially methylated CpGs were identified using Welch’s unequal variance *T* test. *p* Values were adjusted using the p.adjust R function with Bonferroni multiple hypotheses correction method in the stats R package (v3.6.3). Pathway enrichment analysis was performed using the methylglm function from the methylGSA^73^ R package using default settings (v1.4.9). The top 15 enriched gene sets as determined by adjusted *p* value were displayed.

### Reporting summary

Further information on research design is available in the Nature Research Reporting Summary linked to this article.

## Supplementary information

Supplementary Information

Supplementary Tables 1-5

Reporting Summary

## Data Availability

Paediatric POG chordoma gene expression, DNA methylation and small mutation data for both paediatric chordoma patients sequenced at Canada’s Michael Smith Genome Centre can be downloaded from (https://www.bcgsc.ca/downloads/POG_chordoma/). Paediatric POG chordoma genomic and transcriptome sequencing data sets have been deposited at the European Genome-Phenome Archive (patient 1 EGAD00001008012 and patient 2 EGAD00001008013). Gene expression data for the POG570 adult pan-cancer cohort can be downloaded from https://www.bcgsc.ca/downloads/POG570/. POG570 Genomic and transcriptomic data sets have been deposited at the European Genome-Phenome Archive (EGA, https://ega-archive.org/) as part of the study EGAS00001001159. Previously published gene expression data from The Cancer Genome Atlas (TCGA) used in Fig. 1 can be downloaded from https://xenabrowser.net/datapages/. Controlled TCGA data used in Fig. 2 was obtained from dbGaP (http://www.ncbi.nlm.nih.gov/gap). Previously published data by the Therapeutically Applicable Research to Generate Effective Treatments (TARGET) initiative, phs000218, managed by the NCI were obtained from https://portal.gdc.cancer.gov/ and that by the Treehouse Childhood Cancer Initiative were obtained from University of California, Santa Cruz (https://treehousegenomics.soe.ucsc.edu/). All other data supporting the findings of this study are available from the authors upon reasonable request.

## Code availability

The bioinformatics analyses were performed using open-source software, including Burrows–Wheeler alignment tool (v.0.7.6a), CNAseq (v.0.0.6), APOLLOH (v.0.1.1), SAMtools (v.0.1.17), MutationSeq (v.4.3.5), Strelka (v.1.0.6), SNPEff (v.3.2), ABySS (v.1.3.4), TransABySS (v.1.4.10), Chimerascan (v.0.4.5), DeFuse (v.0.6.2), Manta (v.1.0.0), Delly (v.0.7.3), MAVIS (v.2.1.1), STAR (v.2.5.2b), RSEM (v.1.3.0), samtools (v.0.1.17), CIBERSORT (v.1.04), Jaguar (v.2.0.3), MiXCR (v3.0.5) and VDJtools (v.1.1.9). OptiType (v1.3.1), NetMHCPan (v4.0), Geneious (v8.1.4), AID ELISpot (v.7.0), R (v3.6.0) and associated packages: minfi (v1.32.0), stats(v3.6.3), heatmap3 (v1.1.17), NMF (v0.20.2), umap(v0.2.6.0), methylGSA(v1.4.9), novoAlign (v.3.04.06), novo5mC (0.8.9d). Additional processing involved in-house scripts that are available upon request.

## References

[1] Yeter HG, Kosemehmetoglu K, Soylemezoglu F (2019). Poorly differentiated chordoma: review of 53 cases. APMIS.

[2] Hasselblatt M (2016). Poorly differentiated chordoma with SMARCB1/INI1 loss: a distinct molecular entity with dismal prognosis. Acta Neuropathol..

[3] Shih AR (2018). Clinicopathologic characteristics of poorly differentiated chordoma. Mod. Pathol..

[4] Antonelli M (2017). SMARCB1/INI1 involvement in pediatric chordoma: a mutational and immunohistochemical analysis. Am. J. Surg. Pathol..

[5] Jaber OI, Ashhab MA (2019). Metastatic poorly differentiated chordoma: the eyes do not see what the mind does not know. Autops. Case Rep..

[6] Vaddepally RK, Kharel P, Pandey R, Garje R, Chandra AB (2020). Review of indications of FDA-approved immune checkpoint inhibitors per NCCN guidelines with the level of evidence. Cancers.

[7] Geoerger B (2020). Pembrolizumab in paediatric patients with advanced melanoma or a PD-L1-positive, advanced, relapsed, or refractory solid tumour or lymphoma (KEYNOTE-051): interim analysis of an open-label, single-arm, phase 1-2 trial. Lancet Oncol..

[8] Davis KL (2020). Nivolumab in children and young adults with relapsed or refractory solid tumours or lymphoma (ADVL1412): a multicentre, open-label, single-arm, phase 1-2 trial. Lancet Oncol..

[9] Geoerger B (2020). Atezolizumab for children and young adults with previously treated solid tumours, non-Hodgkin lymphoma, and Hodgkin lymphoma (iMATRIX): a multicentre phase 1-2 study. Lancet Oncol..

[10] Bourdeaut F, Thaku MD, Bergthold G, Karski E (2017). Atrt-11. Marked response to atezolizumab in a patient with rhabdoid tumor: a case study from the imatrix-atezolizumab trial. Neuro-Oncol..

[11] Jelinic P (2018). Immune-active microenvironment in small cell carcinoma of the ovary, hypercalcemic type: rationale for immune checkpoint blockade. J. Natl Cancer Inst..

[12] Wu X (2020). Response of metastatic chordoma to the immune checkpoint inhibitor pembrolizumab: a case report. Front. Oncol..

[13] Forrest SJ (2020). Genomic and immunologic characterization of INI1-deficient pediatric cancers. Clin. Cancer Res..

[14] Leruste A (2019). Clonally expanded T cells reveal immunogenicity of rhabdoid tumors. Cancer Cell.

[15] Miao D (2018). Genomic correlates of response to immune checkpoint therapies in clear cell renal cell carcinoma. Science.

[16] Abro B (2019). Tumor mutation burden, DNA mismatch repair status and checkpoint immunotherapy markers in primary and relapsed malignant rhabdoid tumors. Pathol. Res. Pract..

[17] Chun H-JE (2019). Identification and analyses of extra-cranial and cranial rhabdoid tumor molecular subgroups reveal tumors with cytotoxic T cell infiltration. Cell Rep..

[18] Cancer Genome Atlas Research Network (2017). Comprehensive and integrated genomic characterization of adult soft tissue sarcomas. Cell.

[19] Pleasance E (2020). Pan-cancer analysis of advanced patient tumors reveals interactions between therapy and genomic landscapes. Nat. Cancer.

[20] Pan D (2018). A major chromatin regulator determines resistance of tumor cells to T cell-mediated killing. Science.

[21] Newman AM (2015). Robust enumeration of cell subsets from tissue expression profiles. Nat. Methods.

[22] Pender A (2021). Genome and transcriptome biomarkers of response to immune checkpoint inhibitors in advanced solid tumors. Clin. Cancer Res..

[23] Feng, X. et al. Therapeutic implication of genomic landscape of adult metastatic sarcoma. *JCO Precis. Oncol*. 10.1200/PO.18.00325 (2019). 10.1200/PO.18.00325 35100702

[24] Versteege I (1998). Truncating mutations of hSNF5/INI1 in aggressive paediatric cancer. Nature.

[25] Biegel JA (2002). The role of INI1 and the SWI/SNF complex in the development of rhabdoid tumors: meeting summary from the workshop on childhood atypical teratoid/rhabdoid tumors. Cancer Res..

[26] Griss J (2019). B cells sustain inflammation and predict response to immune checkpoint blockade in human melanoma. Nat. Commun..

[27] Treffers LW (2020). IgA-mediated killing of tumor cells by neutrophils is enhanced by CD47-SIRPα checkpoint inhibition. Cancer Immunol. Res..

[28] Samstein RM (2019). Tumor mutational load predicts survival after immunotherapy across multiple cancer types. Nat. Genet..

[29] Goodman AM (2017). Tumor mutational burden as an independent predictor of response to immunotherapy in diverse cancers. Mol. Cancer Ther..

[30] Bolotin DA (2015). MiXCR: software for comprehensive adaptive immunity profiling. Nat. Methods.

[31] Palena C (2007). The human T-box mesodermal transcription factor Brachyury is a candidate target for T-cell-mediated cancer immunotherapy. Clin. Cancer Res..

[32] Tucker JA (2014). Identification and characterization of a cytotoxic T-lymphocyte agonist epitope of brachyury, a transcription factor involved in epithelial to mesenchymal transition and metastasis. Cancer Immunol. Immunother. CII.

[33] DeMaria PJ (2020). A randomized, double-blind, phase II clinical trial of GI-6301 (yeast-brachyury vaccine) versus placebo in combination with standard of care definitive radiotherapy in locally advanced, unresectable, chordoma. J. Clin. Oncol..

[34] Henon C (2019). Long lasting major response to pembrolizumab in a thoracic malignant rhabdoid-like SMARCA4-deficient tumor. Ann. Oncol..

[35] Blay J-Y (2020). 1619O High clinical benefit rates of single agent pembrolizumab in selected rare sarcoma histotypes: first results of the AcSé Pembrolizumab study. Ann. Oncol..

[36] Theruvath J (2020). Locoregionally administered B7-H3-targeted CAR T cells for treatment of atypical teratoid/rhabdoid tumors. Nat. Med..

[37] Terry RL (2020). Immune profiling of pediatric solid tumors. J. Clin. Investig..

[38] Plesca I (2020). Characteristics of tumor-infiltrating lymphocytes prior to and during immune checkpoint inhibitor therapy. Front. Immunol..

[39] Uryvaev A, Passhak M, Hershkovits D, Sabo E, Bar-Sela G (2018). The role of tumor-infiltrating lymphocytes (TILs) as a predictive biomarker of response to anti-PD1 therapy in patients with metastatic non-small cell lung cancer or metastatic melanoma. Med. Oncol..

[40] Cristescu R (2018). Pan-tumor genomic biomarkers for PD-1 checkpoint blockade–based immunotherapy. Science.

[41] Tumeh PC (2014). PD-1 blockade induces responses by inhibiting adaptive immune resistance. Nature.

[42] Coulie PG, Van den Eynde BJ, van der Bruggen P, Boon T (2014). Tumour antigens recognized by T lymphocytes: at the core of cancer immunotherapy. Nat. Rev. Cancer.

[43] Sahin U (2020). An RNA vaccine drives immunity in checkpoint-inhibitor-treated melanoma. Nature.

[44] Migliorini D (2017). First report of clinical responses to immunotherapy in 3 relapsing cases of chordoma after failure of standard therapies. Oncoimmunology.

[45] Mathios D (2015). PD-1, PD-L1, PD-L2 expression in the chordoma microenvironment. J. Neurooncol..

[46] Feng Y (2015). Expression of programmed cell death ligand 1 (PD-L1) and prevalence of tumor-infiltrating lymphocytes (TILs) in chordoma. Oncotarget.

[47] Fujii R (2016). Enhanced killing of chordoma cells by antibody-dependent cell-mediated cytotoxicity employing the novel anti-PD-L1 antibody avelumab. Oncotarget.

[48] Yates AD (2020). Ensembl 2020. Nucleic Acids Res..

[49] Li H, Durbin R (2009). Fast and accurate short read alignment with Burrows-Wheeler transform. Bioinformatics.

[50] Jones SJ (2010). Evolution of an adenocarcinoma in response to selection by targeted kinase inhibitors. Genome Biol..

[51] Ha G (2012). Integrative analysis of genome-wide loss of heterozygosity and monoallelic expression at nucleotide resolution reveals disrupted pathways in triple-negative breast cancer. Genome Res..

[52] Li H (2009). The Sequence Alignment/Map format and SAMtools. Bioinformatics.

[53] Ding J (2012). Feature-based classifiers for somatic mutation detection in tumour-normal paired sequencing data. Bioinformatics.

[54] Saunders CT (2012). Strelka: accurate somatic small-variant calling from sequenced tumor-normal sample pairs. Bioinformatics.

[55] Simpson JT (2009). ABySS: a parallel assembler for short read sequence data. Genome Res..

[56] Birol I (2009). De novo transcriptome assembly with ABySS. Bioinformatics.

[57] Iyer MK, Chinnaiyan AM, Maher CA (2011). ChimeraScan: a tool for identifying chimeric transcription in sequencing data. Bioinformatics.

[58] McPherson A (2011). deFuse: an algorithm for gene fusion discovery in tumor RNA-Seq data. PLoS Comput. Biol..

[59] Chen X (2016). Manta: rapid detection of structural variants and indels for germline and cancer sequencing applications. Bioinformatics.

[60] Rausch T (2012). DELLY: structural variant discovery by integrated paired-end and split-read analysis. Bioinformatics.

[61] Reisle C (2019). MAVIS: merging, annotation, validation, and illustration of structural variants. Bioinformatics.

[62] Cingolani P (2012). A program for annotating and predicting the effects of single nucleotide polymorphisms, SnpEff: SNPs in the genome of *Drosophila melanogaster* strain w1118; iso-2; iso-3. Fly.

[63] Butterfield YS (2014). JAGuaR: junction alignments to genome for RNA-seq reads. PLoS ONE.

[64] Szolek A (2014). OptiType: precision HLA typing from next-generation sequencing data. Bioinformatics.

[65] Jurtz V (2017). NetMHCpan-4.0: improved peptide-MHC class I interaction predictions integrating eluted ligand and peptide binding affinity data. J. Immunol..

[66] Giudicelli V, Chaume D, Lefranc M-P (2005). IMGT/GENE-DB: a comprehensive database for human and mouse immunoglobulin and T cell receptor genes. Nucleic Acids Res..

[67] Cohen CJ, Zhao Y, Zheng Z, Rosenberg SA, Morgan RA (2006). Enhanced antitumor activity of murine-human hybrid T-cell receptor (TCR) in human lymphocytes is associated with improved pairing and TCR/CD3 stability. Cancer Res..

[68] Robinson J, Halliwell JA, McWilliam H, Lopez R, Marsh SGE (2013). IPD-the Immuno Polymorphism Database. Nucleic Acids Res..

[69] Chen Y (2013). Discovery of cross-reactive probes and polymorphic CpGs in the Illumina Infinium HumanMethylation450 microarray. Epigenetics.

[70] Cancer Genome Atlas Research Network (2015). Comprehensive, integrative genomic analysis of diffuse lower-grade gliomas. N. Engl. J. Med..

[71] Cancer Genome Atlas Research Network. (2014). Comprehensive molecular characterization of gastric adenocarcinoma. Nature.

[72] Gaujoux R, Seoighe C (2010). A flexible R package for nonnegative matrix factorization. BMC Bioinforma..

[73] Ren X, Kuan PF (2019). methylGSA: a Bioconductor package and Shiny app for DNA methylation data length bias adjustment in gene set testing. Bioinformatics.

[74] Johann PD (2016). Atypical teratoid/rhabdoid tumors are comprised of three epigenetic subgroups with distinct enhancer landscapes. Cancer Cell.

