# Supplementary material for: Clinical response to nivolumab in an INI1-deficient pediatric chordoma correlates with immunogenic recognition of brachyury
Source: NPJ Precis Oncol. 2021 Dec 20;5:103. doi: 10.1038/s41698-021-00238-4 (PMC8688516; doi:10.1038/s41698-021-00238-4)
Supplement: Supplementary file 1 — Supplementary Information [file 41698_2021_238_MOESM1_ESM.pdf]

Supplementary Information

Supplementary tables

Supplementary table 1. TCGA Sarcoma and POG570 patient identifiers used in Figure 1 and 2

Supplementary table 2. TRA and TRB sequences identified from patient 2 RNA sequencing data using MiXCR as displayed in Figure 4a.

Supplementary table 3. TBXT/brachyury neoantigens predicted using NetMHCPan for patient 1 and 2. Neoantigens were predicted from all three TBXT/brachyury isoforms (TBXT-201, 202 and 203). Patient 1 and patient 2 HLA alleles were determined using OPTItype.

Supplementary table 4. Gene ontology terms associated with differentially methylated CpGs from pediatric chordoma compared to RT subgroups 1-3 and 5 as in Figure 5d.

Supplementary table 5. Whole genome and Whole genome bisulfite sequencing (WBGS) coverage and RNA sequencing read counts for libraries prepared from biopsy and blood samples for patients 1 and 2.

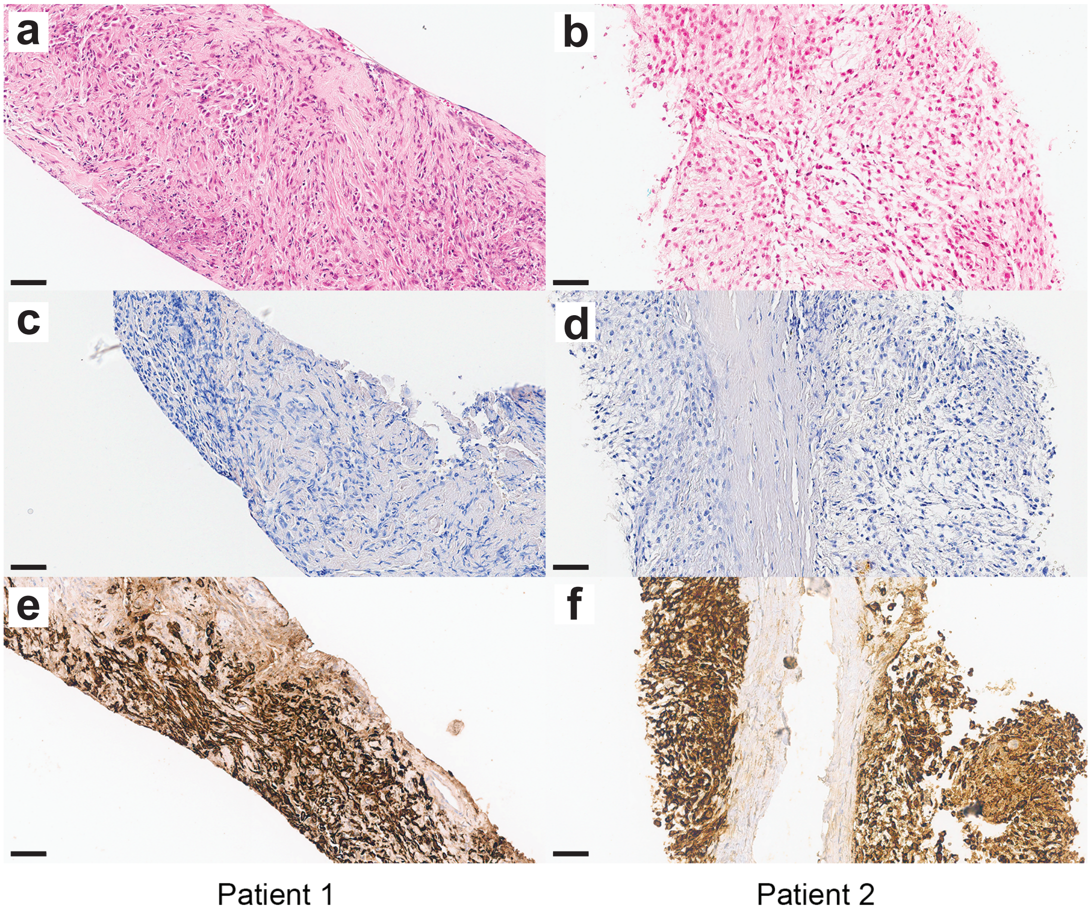

Supplementary Figure 1. H&E, S100 and keratin expression in poorly differentiated pediatric chordoma diagnostic specimens. Formalin- fixed paraffin- embedded (FFPE) sections of the lesional biopsies from patient 1 (a, c, e) and patient 2 (b, d, f) display features consistent with a PDC in these two patients. H&E photomicrographs (a, b) are composed of sheets of epithelioid to spindle cells associated with occasional eosinophilic cytoplasm and vesicular nuclei admixed with fibroblasts and inflammatory cells. Tumour cells are negative for S100 (c, d) and strongly expressed keratin (e, f). These additional features confirm the diagnosis of poorly differentiated chordoma. Original magnification: x 400, scale bars represent 60 μm (a-f).

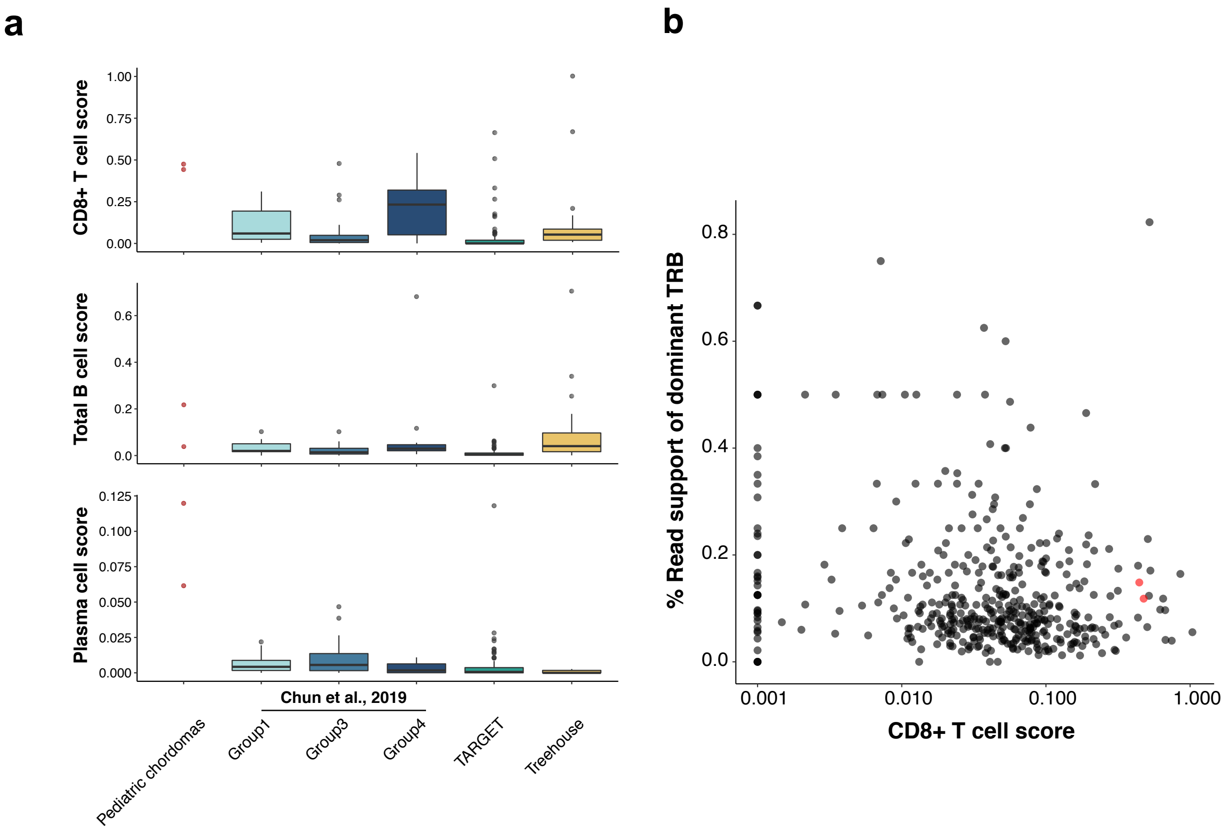

Supplementary Figure 2. Immune microenvironment of poorly differentiated pediatric chordomas compared to pediatric datasets. a) CIBERSORT estimation of CD8+ T cell, B cell and plasma cell abundance from bulk pediatric chordoma RNA sequencing data in pediatric chordomas, pediatric rhabdoid tumours (RT)[17] and the TARGET and Treehouse datasets (https://ocg.cancer.gov/programs/target, https://treehousegenomics.soe.ucsc.edu/). CD8+ T cell scores (top panel): patient 1 = 0.44, patient 2 = 0.48, RT median score = 0.03 (n = 65), TARGET median score = 0.00 (clear cell sarcoma of the kidney n = 13, osteosarcoma n = 87) and Treehouse median score = 0.05 (n = 18, see methods for tumour types). Total B cell scores (middle panel): patient 1 = 0.04, patient 2 = 0.22, RT median score = 0.02, TARGET median score = 0.00, Treehouse median score = 0.04. Plasma cell scores (bottom panel): patient 1 = 0.06, patient 2 = 0.12, RT median score = 0.00, TARGET median score = 0.00 and Treehouse median score = 0.00. Box plots represent median, upper and lower quartiles, and whiskers represent limits of the distributions (1.5-times interquartile range). b) The percentage of read support associated with the most dominant TRB sequence for POG570 (black, n = 447) and pediatric chordomas (red), n =2). The CD8+ T cell score is shown on the x axis.

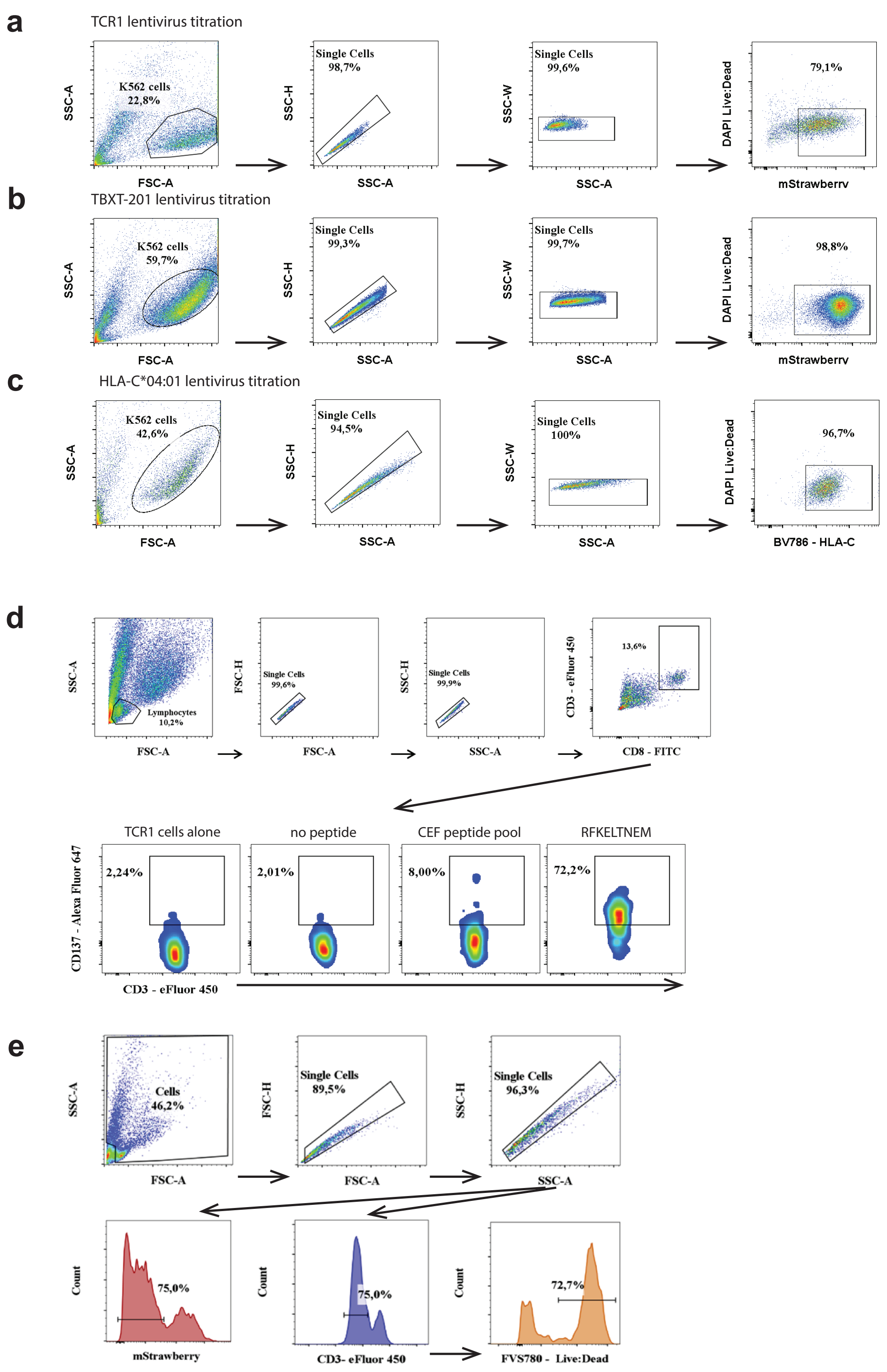

Supplementary Figure 3. Representative lentiviral transduction efficacy, CD137 expression and cell killing flow cytometry assays. a-c) Representative images showing efficacy of K562 cell transduction for a) TCR1, b) TBXT-201 and c) HLA-C*04:01, respectively. TBXT and TCR1 expression was assessed by mStrawberry and HLA allele expression by staining with anti-HLA antibody (see methods, BV786-HLA-C shown here). Images shown here represent typical lentiviral transduction efficacy associated with experiments displayed in Figure 4. d) K562 cells transduced to express HLA-C*04:01, were peptide pulsed with either the CEF peptide pool (1µg/ml), TXBT- HLA-C*04:01 restricted peptide RFKELTNEM (10µg/ml) or HWMKAPVSF (10µg/ml), or were not peptide pulsed and were then co-cultured for 5 hours with donor CD8+ T cells expressing the transgene, TCR1. Gating strategy corresponds to Figure 4f. e) Peptide pulsed, HLA-C*04:01 expressing K562 cells co-cultured with TCR1 T cells as in panel c were assessed for target cell killing by sorting for single cells, followed by staining with anti-CD3 and fixable viability stain (FVS780). The intensity of FVS780 staining for the CD3-negative target cells was plotted to reveal the viable and dead cellular populations. Gating strategy corresponds to Figure 4g.

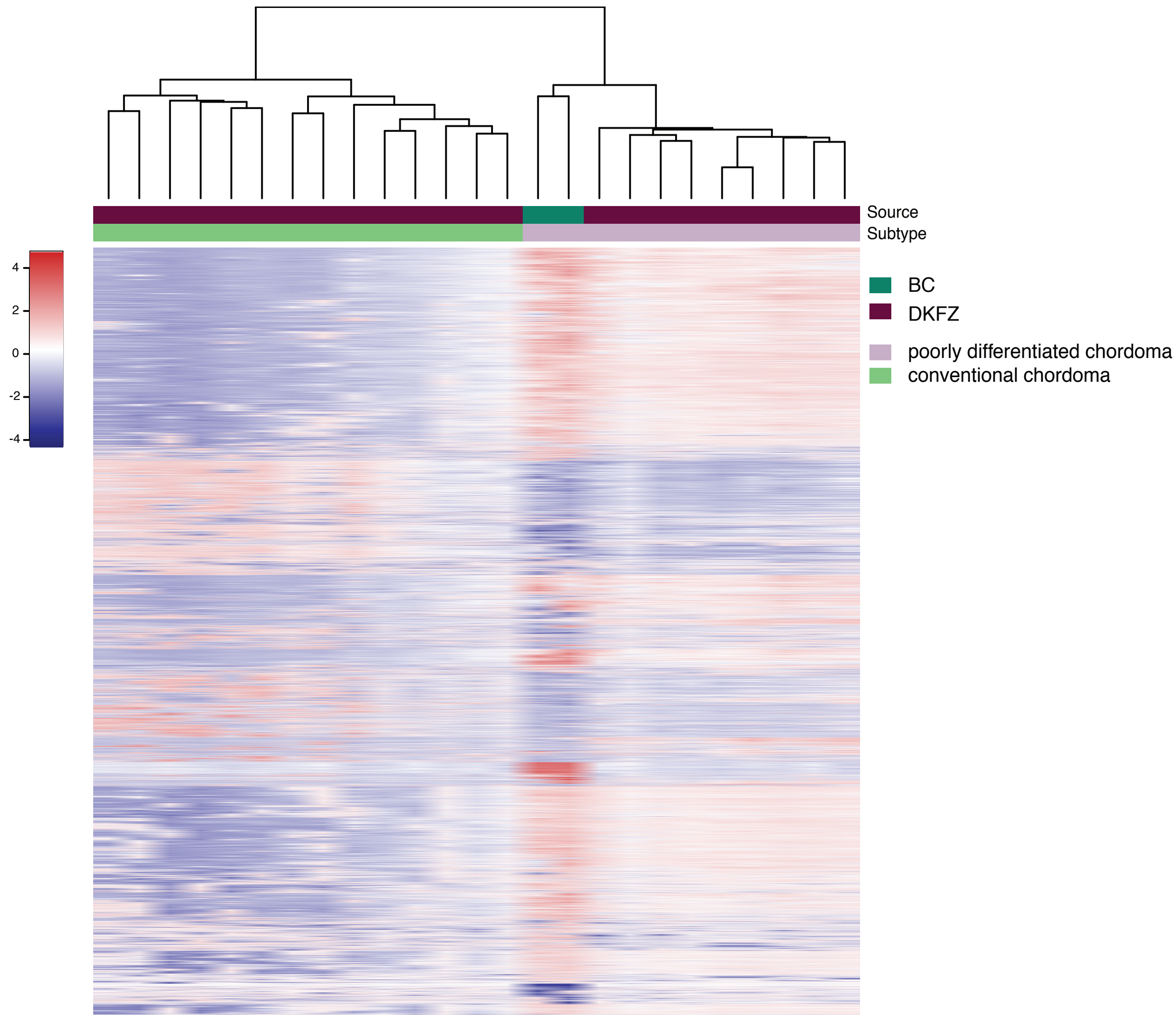

Supplementary Figure 4. DNA methylation clustering of conventional and poorly differentiated pediatric chordomas. Hierarchical clustering of the top 10,000 variably methylated CpG sites across conventional and poorly differentiated chordomas from this study (Williamson et al., n = 2) and Hasselblatt et al.[2] (n = 23).
