# Supplementary material for: Clinical response to nivolumab in an INI1-deficient pediatric chordoma correlates with immunogenic recognition of brachyury
Source: NPJ Precis Oncol. 2021 Dec 20;5:103. doi: 10.1038/s41698-021-00238-4 (PMC8688516; doi:10.1038/s41698-021-00238-4)
Supplement: Supplementary file 3 — Reporting Summary [file 41698_2021_238_MOESM3_ESM.pdf]

nature portfolio

Corresponding author(s): Dr. Marco A. Marra
Last updated by author(s): Sep 15, 2021

Reporting Summary

Nature Portfolio wishes to improve the reproducibility of the work that we publish. This form provides structure for consistency and transparency in reporting. For further information on Nature Portfolio policies, see our Editorial Policies and the Editorial Policy Checklist.

Statistics

For all statistical analyses, confirm that the following items are present in the figure legend, table legend, main text, or Methods section.

n/a	Confirmed	
☐	☒	The exact sample size (*n*) for each experimental group/condition, given as a discrete number and unit of measurement
☐	☒	A statement on whether measurements were taken from distinct samples or whether the same sample was measured repeatedly
☐	☒	The statistical test(s) used AND whether they are one- or two-sided<br>*Only common tests should be described solely by name; describe more complex techniques in the Methods section.*
☒	☐	A description of all covariates tested
☐	☒	A description of any assumptions or corrections, such as tests of normality and adjustment for multiple comparisons
☐	☒	A full description of the statistical parameters including central tendency (e.g. means) or other basic estimates (e.g. regression coefficient) AND variation (e.g. standard deviation) or associated estimates of uncertainty (e.g. confidence intervals)
☐	☒	For null hypothesis testing, the test statistic (e.g. *F*, *t*, *r*) with confidence intervals, effect sizes, degrees of freedom and *P* value noted<br>*Give P values as exact values whenever suitable.*
☒	☐	For Bayesian analysis, information on the choice of priors and Markov chain Monte Carlo settings
☒	☐	For hierarchical and complex designs, identification of the appropriate level for tests and full reporting of outcomes
☒	☐	Estimates of effect sizes (e.g. Cohen's *d*, Pearson's *r*), indicating how they were calculated

Our web collection on statistics for biologists contains articles on many of the points above.

Software and code

Policy information about availability of computer code

Data collection	For collection of WGTA data, sequencing was performed on HiSeqX (HCS version 3.4.0). Bases were called using Illumina Off-line Basecaller v1.9.4, Illumina bcl2fastq v2.17.1.14. Flow cytometry was performed on a FACSymphony cell analyser, and analysed using FlowJo (v10.3)
Data analysis	All analysis used previously published or openly available software. The bioinformatics analyses were performed using open-source software, including Burrows–Wheeler alignment tool (v.0.7.6a), CNAseq (v.0.0.6), APOLLOH (v.0.1.1), SAMtools (v.0.1.17), MutationSeq (v.4.3.5), Strelka (v.1.0.6), SNPEff (v.3.2), ABySS (v.1.3.4), TransABySS (v.1.4.10), Chimerascan (v.0.4.5), DeFuse (v.0.6.2), Manta (v.1.0.0), Delly (v.0.7.3), MAVIS71 (v.2.1.1), STAR (v.2.5.2b), RSEM (v.1.3.0), samtools (v.0.1.17), CIBERSORT (v.1.04), Jaguar (v.2.0.3), MiXCR (v3.0.5) and VDJtools (v.1.1.9). OptiType (v1.3.1), NetMHCPan (v4.0), Geneious (v8.1.4), AID ELISpot (v.7.0), R (v3.6.0) and associated packages: minfi (v1.32.0), stats(v3.6.3), heatmap3 (v1.1.17), NMF (v0.20.2), umap(v0.2.6.0), methylGSA(v1.4.9), novoAlign (v.3.04.06), novo5mC (0.8.9d). Default parameters were used unless otherwise specified. Additional processing involved in-house scripts that are available upon request.

For manuscripts utilizing custom algorithms or software that are central to the research but not yet described in published literature, software must be made available to editors and reviewers. We strongly encourage code deposition in a community repository (e.g. GitHub). See the Nature Portfolio guidelines for submitting code & software for further information.

Data

Policy information about availability of data

All manuscripts must include a data availability statement. This statement should provide the following information, where applicable:

- Accession codes, unique identifiers, or web links for publicly available datasets
- A description of any restrictions on data availability
- For clinical datasets or third party data, please ensure that the statement adheres to our policy

Pediatric POG chordoma gene expression, DNA methylation and small mutation data for both pediatric chordoma patients sequenced at Canada's Michael Smith Genome Centre can be downloaded from (https://www.bcgsc.ca/downloads/POG_chordoma/). Pediatric POG chordoma genomic and transcriptome sequencing datasets have been deposited at the European Genome-Phenome Archive (patient 1 EGAD00001008012 and patient 2 EGAD00001008013). Gene expression data for the POG570 adult pan-cancer cohort can be downloaded from (https://www.bcgsc.ca/downloads/POG570/). POG570 Genomic and transcriptomic datasets have been deposited at the European Genome-Phenome Archive (EGA, https://ega-archive.org/) as part of the study EGAS00001001159. Previously published gene expression data from The Cancer Genome Atlas (TCGA) used in Figure1 can be downloaded from (https://xenabrowser.net/datapages/). Controlled TCGA data used in Figure 2 was obtained from dbGaP (http://www.ncbi.nlm.nih.gov/gap). Previously published data by the Therapeutically Applicable Research to Generate Effective Treatments (TARGET) initiative, phs000218, managed by the NCI was obstained from (https://portal.gdc.cancer.gov/) and by the Treehouse Childhood Cancer Initiative are were obtained from University of California, Santa Cruz (https://treehousegenomics.soe.ucsc.edu/). All other data supporting the findings of this study are available from the authours upon reasonable request.

Field-specific reporting

Please select the one below that is the best fit for your research. If you are not sure, read the appropriate sections before making your selection.

☒ Life sciences ☐ Behavioural & social sciences ☐ Ecological, evolutionary & environmental sciences

For a reference copy of the document with all sections, see nature.com/documents/nr-reporting-summary-flat.pdf

Life sciences study design

All studies must disclose on these points even when the disclosure is negative.

Sample size	We present the data generated from 2 paediatric chordomas sequenced as part of the POG program.
Data exclusions	No data was excluded from this study
Replication	For Figure 2, a minimum of 10 multiplex IHC images were analyzed by 5 different algorithms. The average of all 5 algorithms from all experimental results from each image are included.<br>For Figure 4, Elispot experiments were replicated 3 times, and all experimental results are shown. Flow cytometry analysis of CD137 expression and T cell-mediated cell killing experiments were replicated 4 times.
Randomization	Samples were allocated based on primary tumour diagnosis, and in the case of rhabdoid tumours, based on previously published subgroups in Chun et al.
Blinding	Blinding was not performed in this study. All analyses were performed on groups defined by inherent sample characteristics, specifically tumour type/subtype, which was not assessed by the experimenter and was therefore not subject to bias.

Reporting for specific materials, systems and methods

We require information from authors about some types of materials, experimental systems and methods used in many studies. Here, indicate whether each material, system or method listed is relevant to your study. If you are not sure if a list item applies to your research, read the appropriate section before selecting a response.

Materials & experimental systems

n/a	Involved in the study	
☐	☒	Antibodies
☐	☒	Eukaryotic cell lines
☒	☐	Palaeontology and archaeology
☒	☐	Animals and other organisms
☐	☒	Human research participants
☐	☒	Clinical data
☒	☐	Dual use research of concern

Methods

n/a	Involved in the study	
☒	☐	ChIP-seq
☐	☒	Flow cytometry
☐	☒	MRI-based neuroimaging

Antibodies

Antibodies used	Multiplex IHC: anti-granzyme B (GrB-7, MA1-35461 Thermo Fisher, 1:20), CD8 (C8/144B, 108M-94 Cell Marque, 1:250), CD3 (Sp7, M3074 Spring Bioscience, 1:500), PD-L1 (SP142, M4422 Spring Bioscience, 1:100), PD-1 (NAT105, 315M-94 Cell Marque, 1:100), brachyury/TBXT (EPR18113, ab209665 Abcam, 1:1500), CD79a (SP18, M3182 Spring Bioscience, 1:600), CD68 (KP-1, CM033 Biocare, 1:100)<br><br>Diagnostic pathology: Immunohistochemistry using the Ventana Benchmark automated stainer and the following primary antibodies: pan-keratin (clone AE1/AE3, MS-343-R7 Thermo Scientific, ready to use), BAF47/INI1 (clone 25, 612110 BD Bioscience, 1:100), and S100 (clone 4C4.9, 790-2914 Ventana, prediluted), brachyury (EPR18113, Abcam, 1:2000).<br><br>Flow cytometry and Elispot Assay: HLA-A – PECY5 (Creative Diagnostic, DCABH-4529 1:50), HLA-B – APC (Creative Diagnostic, CABT-BL7610, 1:50) and HLA-C – PE (BD, 566372, 1:50), anti-CD3 (eBiosciences, 16-0037-85, 100 ng/mL) and 100 ng/mL anti-CD28 (BioLegend, 302943, 100 ng/mL), CD8 AF-700 (clone SK1, 344724 BioLegend, 1:1000) CD3-efluor 450 (clone 17A2, 48-0032-82, eBioscience, 1:200), CD137-AF647 (clone 4B4-1, A51019 ThermoFisher, 1:50), CD3-efluor 450 (clone 17A2, 48-0032-82, eBioscience, 1:1000), HLA-A2-FITC (clone BB7.2, 343304 Biolegend, 1:200), IFNγ (1-D1K, 3420-7 Mabtech, 2 µg/ml per well).
Validation	Diagnostic immunohistochemistry was conducted in a certified clinical lab. Multiplex immunohistochemistry antibodies were selected from highly reputable companies and have been used in a number of projects by the Molecular and Cellular Immunology Core. They have been tested on relevant tissues such as tonsil to ensure that they provide the expected staining patterns. Additionally most of the antibodies have been used in complex multicolour immunofluorescent staining and have demonstrated appropriate staining in relation to other markers. The clones selected are highly reported in literature for studies involving immunohistochemical staining.

Eukaryotic cell lines

Policy information about cell lines

Cell line source(s)	Antigen presenting cells: K562 cells (ATCC CCL-243), Lentivirus generation: 293T/17 [HEK 293T/17] (ATCC® CRL-11268TM), CD8+ T cells: Isolated from healthy donor peripheral blood mononuclear cells (Human Peripheral Blood Leukopak, Stemcell technologies) using the Miltenyi MACS human CD8+ isolation kit, as per the manufacturer's protocol (Miltenyi, 130-096-495)
Authentication	All cell lines were sourced from ATCC. Human PBMCs were sourced from StemCell Technologies. Cell lines were not independently authenticated
Mycoplasma contamination	All cell lines tested negative for mycoplasma contamination by PCR
Commonly misidentified lines<br>(See ICLAC register)	HEK293T (clone 17, ATCC) cell line was used for lentivirus production. HEK293T cells are commonly used for production of lentivirus, and contamination with another cell type would not be expected to impact interpretation of the ELISpot assay.

Human research participants

Policy information about studies involving human research participants

Population characteristics	Primary tumour types are specified in the figures and text. When appropriate, we indicate whether tumour samples as adult or paediatric.
Recruitment	Patients are recruited as part of the Personalized Onocogenomics Program at BC Cancer. Patients are diagnosed with advanced disease. Both paediatric chrodoma patients in this study were previously treated with chemotherapy, and had progressive disease.
Ethics oversight	This work was approved and conducted under the University of British Columbia Children's and Women's Research Ethics Board (H13-01640).

Note that full information on the approval of the study protocol must also be provided in the manuscript.

Clinical data

Policy information about clinical studies

All manuscripts should comply with the ICMJE guidelines for publication of clinical research and a completed CONSORT checklist must be included with all submissions.

Clinical trial registration	NCT02155621
Study protocol	https://clinicaltrials.gov/ct2/show/NCT02155621
Data collection	Patients residing in the province of British Columbia were referred to the POG program by their treating oncologist and were selected for functional status, available treatment options and ability to undergo biopsy procedures. Enrollment criteria included locally advanced or metastatic cancer and ECOG 0 or 1. Patients with life expectancy < 6 months were excluded. Selected patients or pediatric patients families were approached for study participation by a POG trained oncologist or study nurse. Samples included in this study were enrolled in the program in 2018.

Outcomes	We report on the clinical response of one patient treated with nivolumab. Response was assessed by Response Assessment in Neuro-Oncology criteria from MRI scans at baseline and after 3 months of nivolumab. MRI scans at cycles 7, 11 and 14 showed gradual progression.

Flow Cytometry

Plots

Confirm that:

☒ The axis labels state the marker and fluorochrome used (e.g. CD4-FITC).

☒ The axis scales are clearly visible. Include numbers along axes only for bottom left plot of group (a 'group' is an analysis of identical markers).

☒ All plots are contour plots with outliers or pseudocolor plots.

☒ A numerical value for number of cells or percentage (with statistics) is provided.

Methodology

Sample preparation

For CD137 assays, TCR1 T cells co-cultured with the APCs (K562 cells transduced with the relevant recombinant HLA allele) at an effector : target ratio of 1:4 for 24 hours before being stained with the CD8 AF-700 (clone SK1, 344724 BioLegend) CD3-efluor 450 (clone 17A2, 48-0032-82, eBioscience), CD137-AF647 (clone 4B4-1, A51019, ThermoFisher), conjugated flow antibodies. Cells and antibodies were incubated at 4ºC for 30 min, then washed by adding 2mL of FACS media (1x D-PBS 14190-144, Gibco with 2% HI-FBS 10082-147, Gibco) to each tube and centrifuging for 10 minutes at 400x g at 4˚C, discarding the supernatant before resuspension in 400µl of cold FACS media.
For cell killing assays, TCR1 T cells were co-cultured with APC's (K562 cells transduced with the relevant recombinant HLA allele) at an effector : target ratio of 4:1 for 4 hours, before being stained with the CD3-efluor 450 (clone 17A2, 48-0032-82, eBioscience) conjugated flow antibody, and the Fixable Viability Dye eFluor 780 (eBioscience 65-0865-14). Cells were stained with the antibodies / viability dye and incubated at 4ºC for 30 min, then washed by adding 2mL of FACS media to each tube and centrifuging for 10 minutes at 400x g at 4˚C, discarding the supernatant before resuspension in 400µl of cold FACS media.
Functional viral titers were established by infection of 5x104 K562 cells (ATCC) with 1, 2, 4, 8, 16, or 32 µL of concentrated viral supernatant. Performed in duplicate, cells were suspended in a final volume of 500µL of sRPMI-1640 media, in the 24-well format. Cells were then incubated for 72 hours, before being washed by adding 2mL of FACS media to each tube and centrifuging for 10 minutes at 400x g at 4˚C, discarding the supernatant before resuspension in 400µl of cold FACS media. For the TCR1 transduced cells, the reporter gene mStrawberry was used to determine transduction. For the creation of the K562 cells expressing the recombinant HLA alleles, the cells were stained with HLA-A Pe-CY7 (Clone X7/43, DCABH-4529, Creative Diagnostic), HLA-B APC (Clone C2.34.3, CABT-BL7610, Creative Diagnostic), or HLA-C (Clone DT-9, BD 747583, BD Biosciences) depending on which recombinant HLA allele the APCs were transduced with.

Instrument

BD FACSymphony cell analyser

Software

Analysed using FlowJo and GraphPad Prism software

Cell population abundance

For a representative experiments involving APC's or target cells (K562-rHLA-C*04:01), peptide pulsed with the RFKELTNEM predicted peptide (Supplementary Figure 3d). Lymphocytes based on the SSC-A/FSC-A gate represented 10.2% of the population, single cells based on FSC-H/FSC-A were 99.6% of the gated Lymphocyte population and SSC-A/SSC-H gated population were 99.9% of the gated FSC-A/FSC-H population. The CD3/ CD8 positive T cells were represented by 13.6% of the SSC-A/SSC-H gated population and of these, 72.2% showed positive CD137 expression, shown in the top CD137/CD3 plot. In contrast, the same experiment with no peptide resulted in 2.0% CD137 positive T cells.
For representative experiments involving cell killing assays (Supplementary Figure 3e), the SSC-A/FSC-A gate, labelled Cells, represented 46.2% of the population. Single cells based on FSC-H/FSC-A represented 89.5% of the SSC-A/FSC-A gated population and the SSC-A/SSC-H gate represented 96.3% of the FSC-H/FSC-A gated population. The histogram of mStrawberry expression, showed that 75.0% of the SSC-A/SSC-H gated cells were mStrawberry negative which is verified by the same percentage of SSC-A/SSC-H gated cells which are CD3 -eFluor 450 negative. Then 72.7% of the CD3 negative cell population, stained positive for FVS780, indicating death of these cells.
For the representative TCR1 lentivirus titration experiments (Supplementary Figure 3a), the FSC-A/SSC-A gate, labelled K562 cells, represented 22.8% of the population, single cells based on SSC-A/SSC-H were 98.7% of the gated K562 cells population, SSC-A/SSC-W represented 99.6% of the SSC-A/SSC-H population, and the mStrawberry/live population represented 79.1% of the SSC-A/SSC-W population and represents K562 cells transduced with 32µl of concentrated TCR1 lentivirus. For the representative TBXT-201 lentivirus titration experiments (Supplementary Figure 3b), the FSC-A/SSC-A gate represented 59.7% of the population, single cells based on SSC-A/SSC-H were 99.3% of the gated K562 cells population, SSC-A/SSC-W represented 99.7% of the SSC-A/SSC-H population, and the mStrawberry/live population represented 98.8% of the SSC-A/SSC-W population and represents K562 cells transduced with 32µl of concentrated TBXT-201 lentivirus. For the representative HLA-C*04:01 lentivirus titration experiments (Supplementary Figure 3c), the FSC-A/SSC-A gate, labelled K562 cells, represented 42.6% of the population, single cells based on SSC-A/SSC-H were 94.5% of the gated K562 cells population, SSC-A/SSC-W represented 100% of the SSC-A/SSC-H population and the BV786-HLA-C/live population represented 96.7% of the SSC-A/SSC-W population and represents K562 cells transduced with 32µl of concentrated HLA-C*04:01 lentivirus.

Gating strategy

For the assessment of CD137 expression by activated T cells (Supplementary Figure 3d), after debris and non-lymphocytes were eliminated by FSC-A vs. SSC-A gating, cell doublets and clumps were then eliminated by FSC-H vs. FSC-A gating followed by SSC-A vs. SSC-H gating. The CD3 and CD8 positive cells were determined by gating cells positive for both the eFluor 450 - CD3Ab, captured using the 405nm laser with the 450/50 filter and FITC-CD8Ab, captured by the 488nm laser with the 515/20

filter. Following this, to determine CD137 expression by the CD3+ CD8+ T cells, the eFluor 450 - CD3Ab was plotted against the Alexa Fluor 647 - CD137Ab, which was captured by the 637nm laser with the 670/30 filter. Finally, while not included in the gating strategy, we did use the 561nm laser, with the 610/20 filter to capture mStrawberry expression by the TCR1 positive cells only to determine what impact, if any, the mStrawberry reporter gene would have on the above mentioned antibody flow panel.
For the cell killing assays (Supplementary Figure 3e), after debris was eliminated by FSC-A vs. SSC-A gating, cell doublets and clumps were then eliminated by FSC-H vs. FSC-A gating followed by SSC-A vs. SSC-H gating. To separate out the two cells in the co-culture, we used the eFluor 450 - CD3Ab (captured using the 405nm laser and the 450/50 filter) to select out the effector TCR1 T cells. This was verified by looking at the percentage of SSC-A vs. SSC-H gated cells which also express the mStrawberry reporter gene, expressed by transduced TCR1 T cells (captured using the 561nm laser, with the 610/20 filter). To measure the death of the CD3 negative target cells, we measured them using the Fixable Viability Stain 780 (FVS780) and plotted them on a histogram showing peaks for the live and dead cell populations.
For lentiviral transduction, we used the mStrawberry reporter gene in the TCR1 and the TBXT transfer vectors to confirm successful transduction of K562 cells (ATCC) (Supplementary Figure 3a and b). After debris was eliminated by FSC-A vs. SSC-A gating, cell doublets and clumps were eliminated by SSC-H vs. SSC-H gating followed by SSC-A vs. SSC-W gating. The gated cells were then analysed for mStrawberry expression (captured using the 561nm laser, with the 610/20 filter) and checked for viability using DAPI (captured using the 405nm laser with the 450/50 filter). In the case of the HLA-expressing APCs the expression of the recombinant HLA-A, HLA-B, and/or HLA-C allele (Supplementary Figure 3c), after debris was eliminated by FSC-A vs. SSC-A gating, cell doublets and clumps were then eliminated by SSC-A vs. SSC-H gating followed by SSC-A vs. SSC-W gating. For the specific HLA expression we used the HLA-A Pe-CY7 (captured using the 561nm laser, with the 780/60 filter), HLA-B APC (captured using the 637nm laser, with the 670/30 filter), and/or the HLA-C BV786 (captured using the 405nm laser, with the 780/60 filter) conjugated antibodies, depending on which HLA the APCs were transduced with. Furthermore, we checked cell viability using DAPI (captured using the 405nm laser with the 450/50 filter). All the collected flow data was analysed using Flow Jo v10, for windows.

☒ Tick this box to confirm that a figure exemplifying the gating strategy is provided in the Supplementary Information.

Magnetic resonance imaging

Experimental design

Design type: NA

Design specifications: NA

Behavioral performance measures: NA

Acquisition

Imaging type(s): structural

Field strength: 3 Tesla

Sequence & imaging parameters: Figure 3 top left: Sagittal contrast-enhanced fat-suppressed 2D T1 spin echo sequence; slice thickness 4 mm (0.8 mm gap); TE/TR 6/800; flip angle 75; FOV 220; matrix 256/192
Figure 3 top middle: Axial contrast-enhanced fat-suppressed 2D T1 spin echo sequence; slice thickness 4 mm (0.8 mm gap); TE/TR 6/800; flip angle 75; FOV 220; matrix 256/192
Figure 3 bottom left: Sagittal contrast-enhanced 3D T1 spoiled gradient echo sequence; slice thickness 1 mm (0 mm gap); TE/TR 2.26/1800; inversion time 900; flip angle 8; FOV 256; matrix 256/224
Figure 3 bottom middle: Axial contrast-enhanced fat-suppressed 2D T1 spin echo sequence; slice thickness 4 mm (1 mm gap); TE/TR 12/750; flip angle 160; FOV 200; matrix 320/240

Area of acquisition: whole brain

Diffusion MRI ☐ Used ☒ Not used

Preprocessing

Preprocessing software: NA

Normalization: NA

Normalization template: NA

Noise and artifact removal: NA

Volume censoring: NA

Statistical modeling & inference

Model type and settings: NA

Effect(s) tested	NA

Specify type of analysis: ☐ Whole brain ☐ ROI-based ☐ Both

Statistic type for inference (See Eklund et al. 2016)	NA
Correction	NA

Models & analysis

n/a	Involved in the study
☒	☐ Functional and/or effective connectivity
☒	☐ Graph analysis
☒	☐ Multivariate modeling or predictive analysis
